# RebiQoL: A randomized trial of telemedicine patient support program for health-related quality of life and adherence in people with MS treated with Rebif

**DOI:** 10.1371/journal.pone.0218453

**Published:** 2019-07-05

**Authors:** Anne-Marie Landtblom, Dimitri Guala, Claes Martin, Stefan Olsson-Hau, Sara Haghighi, Lillemor Jansson, Sten Fredrikson

**Affiliations:** 1 Department of Neuroscience/Neurology, Uppsala University, Uppsala, Sweden; 2 Merck AB, Stockholm, Sweden; 3 Department of Biochemistry and Biophysics, Stockholm University, Science for Life Laboratory, Solna, Sweden; 4 Neurology Unit, Division of Internal Medicine, Danderyd Hospital, Karolinska Institutet, Stockholm, Sweden; 5 Department of Neurology, Skåne University Hospital, Malmö, Sweden; 6 Department of Neurology, Motala Hospital, Motala, Sweden; 7 Department of Neurology, Academic Hospital, Uppsala, Sweden; 8 Department of Clinical Neuroscience, Karolinska Institutet, Stockholm, Sweden; IRCCS E. Medea, ITALY

## Abstract

RebiQoL was a phase IV multicenter randomized study to assess the impact of a telemedicine patient support program (MSP) on health-related quality of life (HRQoL) in patients with relapsing-remitting MS (RRMS) being administered with Rebif with the RebiSmart device. The primary endpoint was to assess the impact of MSP compared to patients only receiving technical support for RebiSmart on HRQoL at 12 months, using the psychological part of Multiple Sclerosis Impact Scale (MSIS-29), in patients administered with Rebif. A total of 97 patients diagnosed with RRMS were screened for participation in the study of which 3 patients did not fulfill the eligibility criteria and 1 patient withdrew consent. Of the 93 randomized patients, 46 were randomized to MSP and 47 to Technical support only. The demographic characteristics of the patients were well-balanced in the two arms. There were no statistical differences (linear mixed model) in any of the primary (difference of 0.48, 95% CI: -8.30–9.25, p = 0.91) or secondary outcomes (p>0.05). Although the study was slightly underpowered, there was a trend towards better adherence in the MSP group (OR 3.5, 95% CI 0.85–14.40, p = 0.08) although not statistically significant. No unexpected adverse events occurred. This study did not show a statistically significant effect of the particular form of teleintervention used in this study on HRQoL as compared to pure technical support, for MS patients already receiving Rebif with the RebiSmart device.

**Trial Registration**: ClinicalTrials.gov: NCT01791244.

## Introduction

The management of patients with multiple sclerosis (MS) treatment has changed substantially during the last 25 years: immunomodulating drugs have been developed and the knowledge of suitable strategies for patient care, including strategies on health-related quality of life (HR-QoL) and fatigue has increased [1].

Furthermore, the use of the internet to deliver web-based interventions to patients for optimizing the best care has rapidly increased during the last decade. Benefits of integrating telecare and web-based interventions into routine health service are today discussed with the emphasis on integrating the aspects of quality of life [2].

Examples from other fields of medicine than MS show interesting results. Patients with type 2 diabetes monitored by telecare and receiving web-based interventions demonstrate significant improvement in glycemic control [3]. In another web-based study, improvement regarding depressive symptoms, anxiety and psychological well-being was seen in a group of university students [4]. Several studies also demonstrate benefits of web-based support to caregivers of patients with chronic disease [5].

Modern technology has provided new tools for patient support with telesupport and web-based interventions, demonstrating that patient care can move out from the hospital and be managed at a distance. Web-based interventions have been tried in MS with different aims. For example, web-based medical and health information has been adapted for shared decision making in Germany and Italy [6]. Another example shows a phone counseling program to improve fatigue and depression with home-based monitoring and physical activity intervention [7].

A pilot study by Zissman et al [8] found that people who were supported by a home telecare model showed improvement in their disease severity and had an increased level of satisfaction with provided care, compared to the group of MS patients receiving standard care [9].

This early example of Zissman in 2012 [8], with the telecare program from the hospital-based health team, attracted positive expectancies because of its results. However, the publication that followed left room for interpretation of the observed efficacy, according to Porter and Thompson.

A review by Tallner in 2016 [10] investigates the role of tele-intervention regarding physical activity in MS, i.e. online exercise programs, and demonstrates a major problem of decreasing participation and adherence over time, although the patient satisfaction was high [11]. A suggestion of developing the method using gamification, was given by the authors.

In the MS arena, it has been claimed that the Department of Veterans’ Affairs leads the development of telesupport for MS. Studies from the US have shown that American MS patients spend much more time on the internet than the average person, giving web-based interventions a potential weight [9].

Additional research findings on possible tele- or web-interventions in MS give support to self-care. Examples include the importance of physical training [12], the importance of stress management to cope with fatigue and heat sensitivity [13–15] and the importance of tobacco smoking on the risk of developing disability, i.e. progressive disease [16]. These examples give substantial reason to support self-care in these different domains. Furthermore a meta-analysis of tele-interventions using psychological support on psychosocial issues in MS showed positive impact [9].

Cost-effective care through web-based interventions and telemedicine are today often requested by MS patients. The aim of the current study (RebiQol) was to investigate one such approach; to assess the impact of a telemedicine patient support program My Support Plus (MSP) on health-related quality of life in patients with relapsing-remitting MS (RRMS) being administered Rebif with the RebiSmart device. Our hypothesis was that the addition of MSP on top of the plain technical support would have a positive impact on the Health-Related quality of life (HRQoL) of people with MS treated by Rebif with the RebiSmart.

## Materials and methods

### Methodology

The RebiQol study was a randomized, comparative, multicentre study. The aim of the study was to assess the impact of the patient support program, My Support Plus (MSP), on Health-Related quality of life (HR-QoL) and adherence in RRMS patients administered Rebif using the RebiSmart device, compared to patients only receiving technical support for RebiSmart. The RebiSmart device is an electronic device for auto-injection of Rebif used in routine clinical practice, which also records time of injection and thus adherence.

The main study hypothesis was that the addition of MSP to plain technical support would result in improvement of HRQoL of people with MS, using RebiSmart. Therefore the primary objective of the study was to assess the impact of MSP, on HRQoL at 12 months, using the psychological scale of the, MS Impact Scale based on 29 items (MSIS-29) [17], in patients administered Rebif with the RebiSmart device compared to patients only receiving technical support for RebiSmart (including assistance with device use and maintenance).

The secondary objectives of the study included assessment of impact of MSP on HRQoL at 6 and 12 months using MSIS-29 (full version) and EQ5D-5L [18] scales as well as the psychological part of the MSIS-29 scale at 6 months. Further the impact of MSP at 6 and 12 months was studied on fatigue using the Fatigue Severity Scale (FSS) [19] and the Modified Fatigue Impact Scale (MFIS). The effects of MSP on adherence, defined as the proportion of patients with <10% missed injections and psychological wellbeing on the Hospital Anxiety and Depression Scale (HADS) [20] of the patients was also assessed at 6 and 12 months. Additional objectives included the study of effects of MSP at 12 months from baseline on working ability (S1 File), on patient and health care personnel satisfaction (S1 File), as well as on the number of reported adverse events at 12 months.

The primary outcome of the study was change from baseline in MSIS-29 psychological score at month 12. Secondary outcomes included change from baseline to month 6 in MSIS-29 psychological score and change from baseline to months 6 and 12 in: MSIS-29 total score, EQ5D-5L summary score, and visual analogue scale, FSS score, MFIS score, and index, and HADS score. Additional secondary outcomes were percentages of subjects with treatment adherence at month 6 and 12 and subjects with AEs at month 12 as well as the numbers of subjects with: working ability at month 12, response to the Life style, patient satisfaction and health-care personnel satisfaction questionnaires and Lifestyle goals for MSP at month 12.

### Description of the intervention

MSP is a patient support program provided by the independent vendor, Health Solutions. The program consists of 7 phone calls (week 1, 3, 7, 11, 19, 31 and 43), 3 text messages (week 5, 9 and 21) and 9 e-mails (day 0, 5, 10, 49, 110, 140, 170, 190, 245 and 343) spread out over the course of the study. Support to the patients is provided by an experienced MS nurse at Health Solutions, who acts as a support coach for the patients. The topics for the calls are:

- Registration and information about the patient support program
- Management of the RebiSmart device and treatment with Rebif
- MS and the treatment effect of Rebif
- Exercise and physical activity
- Adherence to Rebif treatment
- Motivation
- Summary of the past year that and of the activities of the patient support program

The MS nurse follows a detailed manuscript during the calls but there are also opportunities for the patients to speak freely regarding any concerns they might have considering their lives with MS. The e-mails and text messages are evenly distributed between the calls. The text messages act as short reminders of the recent conversation and the e-mails contribute with more in-depth information on the same topics mentioned above. During the entire 12-month period the patients are also presented with the possibility of contacting the coach nurse via telephone during daytime on weekdays regarding any questions they might have. A web-based health journal was also offered at www.minsupport.nu where the patients could track their progress regarding physical activity and get advice on managing the RebiSmart device and the Rebif treatment.

### Patients & assessments

To be included into the study, patients had to fulfil all of the following inclusion criteria; aged 18 or older, a diagnosis of relapsing remitting MS (RRMS) according to the revised McDonald Criteria (2010), treatment with Rebif 22 mcg or 44 mcg subcutaneously (SC) three times a week (tiw) in accordance with the Summary of Product Characteristics, Rebif administered by the RebiSmart device and provide a signed informed consent form (ICF).

Patients fulfilling any of the following criteria were not eligible for the study: having received any components, except for technical support, of MSP prior to study entry; difficulty reading and/or understanding Swedish; having a mental condition rendering the patient unable to understand the nature, scope and possible consequences of the study; and/or evidence of an uncooperative attitude; no access to computer; or participation in another clinical study.

**Fig 1:**
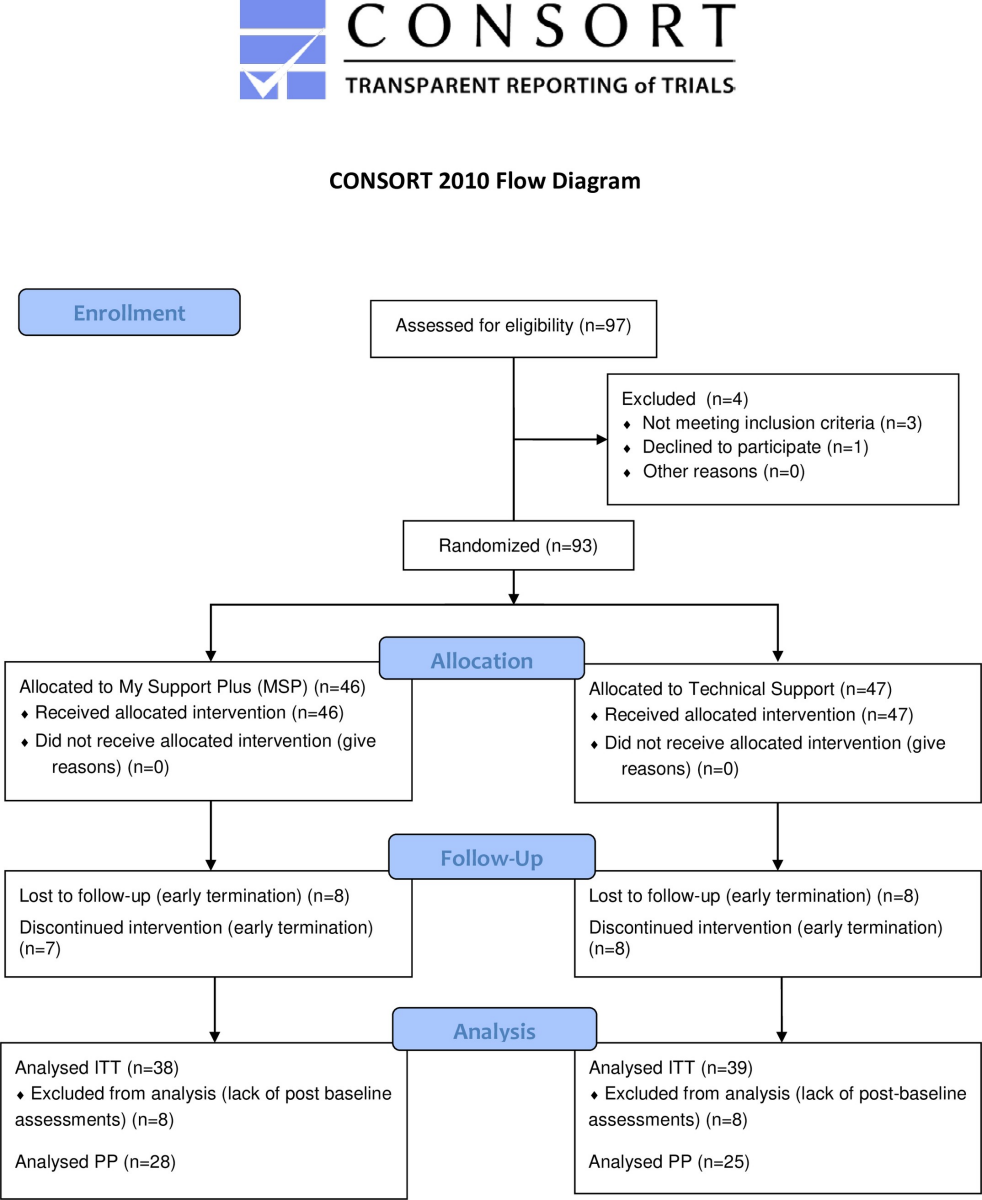
Patient flow. The outline of the selection and randomization flow of patients in the study. Intent to treat (ITT), Per protocol (PP).

**Fig 2:**
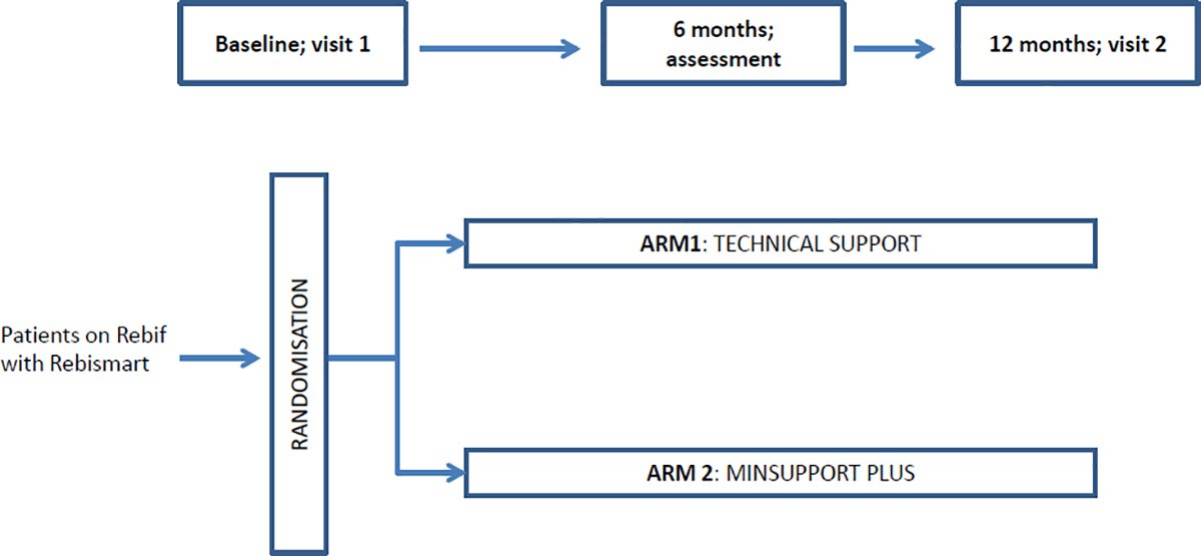
Trial design. Patients on Rebif using Rebismart were randomized to the two treatment arms at the Baseline visit. The study contained two additional assessment timepoints, at 6 and 12 months post baseline, where patients were assessed in accordance with the protocol (S1 File).

The first patient was included in the study February 2013 and the last patient exited the study in December 2015. The recruitment period was extended from 12 to 23 months. The trial was conducted at 11 MS centers in Sweden. A total of 97 patients were screened (Fig 1). The planned study period was 12 months. The trial was designed according to Fig 2.

Patients were randomized into two groups in a blinded one-to-one fashion (S1 File). Both groups had follow-up visits at conventional team-based MS-clinics. The first visit was an investigator-led visit were the patient characteristics were recorded, all study questionnaires were completed by the patient while still in the clinic, and the patient was randomized in a one-to-one fashion to receive technical support or MSP. At the 6-month assessment the patients in both arms received questionnaires, by post, from the external vendor in charge of providing MSP and technical support, for completion and return to the vendor prior to the next visit. Before Visit 2 the patient had to complete a new set of questionnaires once again provided by the external vendor and return the questionnaires for evaluation. Patients in in the intervention arm also filled in a web-based lifestyle questionnaire. Working ability was recorded in the electronic case report form (eCRF) and adherence data was downloaded from the RebiSmart device.

### Statistical methods

Assuming a group difference of 14 points (Arm 2 superior to Arm 1) and a standard deviation of 25 points on the primary endpoint a total of 104 patients were deemed to be required for 80% power and a 5% two-sided significance level t-test with a randomization ratio of 1:1. In order to allow for a 20% drop-out rate, 130 patients were planned for inclusion.

Descriptive summary statistics or frequency counts of demographic and baseline data were presented by treatment arm and overall for the intent to treat (ITT) and per protocol (PP) populations. The PP population was defined as individuals who had completed all scheduled study visits, while the ITT referred to those with data from at least two study visits. The MSIS-29 scale used to assess the impact of MS on the HRQoL consists of 29 items each scored 1–4 with higher scores meaning an increased impairment of HRQoL for the corresponding item. MSIS-29 is divided in a Physiological subscale consisting of items 1–20 and a psychological part consisting of the remaining items 21–29. The observed score *S_O_* is the sum of individual scores ∑*_i_s_i_* and can be normalized to a range of between 0 and 100 using the following equation:

$$S_{N}=\frac{100*{(S}_{O}-S_{min})}{S_{max}-S_{min}}$$ (1)

Where *S_N_* is the normalized score and *S_min_*, and *S_max_* are the minimum and maximum total scores, respectively. For the Psychological part of MSIS-29 the *S_min_* = 9 and *S_max_* = 36 making the *S_N_* = 100(*S_O_*−9)/(36−9).

The primary endpoint was analysed using a linear mixed model (with SAS default VC covariance structure) with the change from baseline to month 6 and month 12 as dependent variable and group, randomization factors, time and baseline value of the dependent variable as fixed factors. The patient specific intercept was included as a random factor. The difference between groups at month 6 and month 12 were presented along with 95% confidence intervals. The 12 months was the primary endpoint. Numeric secondary endpoints were analysed using the same method as for the primary endpoint. Dichotomized endpoints were analysed at month 6 and month 12 separately using a logistic regression. The results are presented as odds ratios with 95% confidence intervals. There was no imputation of missing data.

All statistical analyses were performed using SAS (Version 9.2 or higher). All confidence intervals were two-sided at the 95% level.

The study was approved by the Regional Ethics Committee of Linköping (dnr 2012/347-31).

## Results

A total of 97 patients were screened for participation in the study. Three had already received components of MSP and one withdrew consent. The sample size was originally calculated to include 130 patients to have 104 evaluated patients. Despite the extended recruitment period, only 93 patients were randomized (Fig 1). Of the 93 remaining patients, 46 were randomized to MSP and 47 to Technical support (Fig 1).

The Intent to treat (ITT) population was defined as patients having at least one post-baseline assessment and the per protocol (PP) population required all assessment to be present. Sixteen (16) patients, 8 in each arm, were excluded from the ITT population due to lack of post-baseline assessments because of early termination (Fig 1). The excluded patients in the TS arm were older, but in general similar in their baseline characteristics to the MSP patients.

The demographic characteristics of the patients were well balanced in the two arms (Table 1).

**Table 1:** Baseline demographic characteristics.

	MSP	TS
Age (years)		
n/n_missing	38/0	39/0
Mean (±SD)	41 (13.2)	38 (10.9)
Median	42	36
Min,Max	20,72	18,64
Sex–N (%)		
Male	14 (37%)	15 (38%)
Female	24 (63%)	24 (62%)
EDSS score–N (%)		
>4	4 (11%)	1 (3%)
< = 4	34 (89%)	38 (97%)
Education–N (%)		
Elementary school	3 (8%)	6 (15%)
High school	19 (50%)	23 (59%)
University	15 (39%)	10 (26%)
Missing	1 (3%)	0 (0%)

MSP, My Support Plus; TS, Technical Support, SD, standard deviation.

The point estimate for the difference (adjusted for pre-specified covariates) between the two arms, in the primary outcome variable was 0.48 with a 95% confidence interval (-8.3;9.3) (Table 2). The observed difference was not statistically significant (p = 0.91). Examination of within-person changes were also inconclusive.

**Table 2:** Change from baseline to 12 months in the MSIS-29 psychological scale, 9 questions (ITT population).

Visit	n	MSP Mean (SD)	n	TS Mean (SD)	Estimated support arm difference (95% CI)	p-value
Baseline	38	35.19 (24.38)	39	30.48 (20.94)		
Month 12	38	32.75 (25.27)	39	27.45 (20.63)		
Change from baseline to month 12	38	-2.44 (19.38)	39	-3.04 (19.76)	0.48 (-8.30–9.25)	0.9148

MSIS-29, Multiple Sclerosis Impact Scale 29; ITT, intent to treat; MSP, My Support Plus; TS, Technical Support, SD, standard deviation; CI, confidence interval. Test of support arm effect based on the linear mixed model, with baseline value, time, EDSS at baseline and sex as fixed factors.

### Secondary endpoints

The result of the secondary efficacy analysis is presented in Table 3. There were no statistically significant differences between the intervention arms in any of the quality of life related evaluations (MSIS-29, EQ5D-5L, FSS, MFIS and HADS) from baseline to 12 months. Further secondary endpoints included working ability, patient satisfaction and health care personnel satisfaction, none of which could demonstrate a statistically significant difference between the two groups.

**Table 3:** Change from baseline to 12 months in secondary variables (ITT population).

Visit	n	MSP Mean (SD)	n	TS Mean (SD)	Estimated difference (95% CI)	p-value
A. MSIS-29 full scale						
Change from baseline to month 12	38	1.00 (11.34)	39	0.00 (10.45)	0.91 (-3.88–5.70)	0.7076
B. EQ5D-5L Summary Score						
Change from baseline to month 12	38	0.05 (2.51)	39	-0.08 (1.86)	0.06 (-0.87–0.98)	0.9065
C. Fatigue Severity Scale (FSS)						
Change from baseline to month 12	38	0.13 (0.89)	39	0.08 (1.09)	0.02 (-0.41–0.45)	0.9197
D. Fatigue Impact Scale (MFIS)						
Change from baseline to month 12	37	4.47 (12.42)	36	2.12 (12.68)	1.99 (-3.79–7.76)	0.4976
E. Depression and Anxiety (HADS)						
Change from baseline to month 12	38	-0.42 (3.48)	39	-0.18 (3.26)	-0.11 (-1.59–1.37)	0.8855

ITT, intent to treat; MSP, My Support Plus; TS, Technical Support, SD, standard deviation; CI, confidence interval; MSIS-29, Multiple Sclerosis Impact Scale 29. Test of support arm effect based on the linear mixed model, with baseline value, time, EDSS at baseline and sex as fixed factors.

### Analysis of adherence to treatment

Adherence to treatment between the two intervention arms demonstrated an odds ratio of 3.50 (95% CI 0.85–14.40) in favor of the MSP group (Table 4). The difference was however, not statistically significant (p = 0.08).

**Table 4:** Adherence (ITT population; subgroup of patients with full intervention, post-hoc analysis).

Time	n	MSP n (%)	n	TS n (%)	Odds ratio (95% CI)	p-value
Month 12	16	25 (84.21)	35	21 (60.00)	3.50 (0.85–14.40)	0.0831

ITT, intent to treat; MSP, My Support Plus; TS, Technical Support, SD, standard deviation; CI, confidence interval. Test of support arm effect based on logistic regression model, with baseline value, EDSS at baseline and sex as fixed factors.

For the ITT population at 12 months, the proportion of “adherent” (having <10% missed injections) patients was 54% and 66% in the TS and MSP groups, respectively. The proportion of “non-adherent” patients (having >10% missed injections) was 67% and 33% in the TS and MSP groups, respectively.

The data from the 6-month visit showed similar results for all the assessed parameters (S3 File).

### Adverse events

The adverse events (AE) of beta-interferon-1a are previously well described [21]. In this study a total of 79 AEs were reported by 50 (54%) out of 93 patients. One (1.2%) of the 79 AEs was assessed as severe, 31 (39.2%) as moderate and 47 (59.5%) as mild in intensity. Most of the AEs clustered in SOC ‘General disorders and administration site conditions’ (17 AEs) and ‘Nervous system disorders’ (19 AEs). The most common AE was hepatic enzyme increased (7 AEs).

In total there were 3 serious adverse events (SAE) in the study (1 pneumonia and 2 urinary tract infections). No deaths occurred.

## Discussion

The current study failed to show any beneficial effect of the MSP patient support program compared to pure technical support, on HRQoL, when MSP was administered during 1 year to MS patients currently treated with subcutaneous interferon beta 1a and followed per standard clinical practice at conventional MS outpatient clinics.

Several reasons can be attributed to the lack of observable effect in this study. The fact that we were unable to recruit the targeted population, due to slow recruitment, resulted in a relatively low (93) number of randomized patients, reducing the power of the study dramatically, making it challenging to identify a significant effect. Although 77 patients (83%) completed the 6-month follow up only 53 patients (57%) completed the final study assessment. Patients that dropped out of the study either discontinued the treatment or were lost to follow up. The observed attrition could be attributed both to the requirement for patients to mail their completed questionnaires ahead of their 6- and 12-month assessments, and to the fact that other treatment options were becoming available on the Swedish market during the study. Since the number of patients in both the ITT and PP arms was almost identical in both treatment arms, there is little to suggest that the intervention had any effect on the attrition rate. For the remaining evaluable patients, the 12-month study follow up period may have been too short to produce any significant differences between the treatment arms. However, since no trends, favoring the intervention arm, were observed in the data, the lack of power was probably not the main reason for lack of effect.

A potential reason for no observable difference between the study groups may be that the traditional hospital-based outpatient care in its present form in Sweden already supports the patients in such an effective way, that additional efforts in terms of external nurse support have little to add. Less likely, but still probable is that the actual format of the evaluated patient support program (PSP) with lifestyle goals and MS nurse support may have been inadequate, where another PSP in its place could have yielded significant results.

Another reason may be that the actual concept of the study had flaws that prevented observation of positive effect. The fact that both the intervention group (MSP) and the control group (TS) were already on active disease modifying treatment in the form of Rebif, with documented beneficial effect for MS patients [22,23] should also be considered. Perhaps the patients already had sufficient control of their disease that the added value of a PSP was not enough to produce a significant beneficial effect.

The only potential difference between the study arms was seen for patients’ adherence to treatment. This would be expected since the most common reason for lack of adherence is forgetfulness [24], and the patients in the MSP group received several reminders to take their medication during the program. However, longer observation periods and larger patient groups are needed in order to see clinical effects of adherence [21], both unavailable in this study.

The MS care in Sweden has during the last 20 years been structured with a focus on the MS team. The center of the team is the MS nurse, being the “spider in the web” coordinating different interventions and support to the MS patient. This concerns a complex knowledge and experience of MS patients who may suffer from a variety of symptoms that demand different strategies. Such knowledge that during the latest decades has evolved, in particular from nursing sciences, regarding fatigue, bladder and bowel symptoms, sexual and cognitive dysfunction, as well as psychosocial issues in MS, has consequently been used to form pedagogic and medical support programs for the MS patients. In Sweden, there is an MS Nurse Organization that was developed in the 1990’s, and that has played an important role in the development of high skills in MS care.

The background sketched here, gives a potential role for easily accessed, cost-effective tele- and internet-based interventions centered outside the MS clinic, which puts an emphasis to research that can evaluate and certify such strategies. However, our results failed to show convincing, additive effects on HRQoL of MS patients in the context of conventional treatment at hospital-based MS clinics.

## Supporting information

S1 File Consort checklist. (PDF)

S2 File Clinical study protocol. (PDF)

S3 File Rebiqol Clinicaltrials.gov report. (PDF)
