# Supplementary material for: RebiQoL: A randomized trial of telemedicine patient support program for health-related quality of life and adherence in people with MS treated with Rebif
Source: PLoS One. 2019 Jul 5;14(7):e0218453. doi: 10.1371/journal.pone.0218453 (PMC6611587; doi:10.1371/journal.pone.0218453)
Supplement: S1 File — (PDF) [file pone.0218453.s001.pdf]

Clinical Trial Protocol

**Trial Protocol Number**	**EMR 200136-560**
**Title**	**RebiQoL:** A phase IV multicenter randomized study to assess the impact of a patient support program (MinSupport Plus) on health related quality of life (HR**QoL**) and adherence in patients with relapsing remitting multiple sclerosis administered **Rebi**f® with the RebiSmart device.
**Phase**	**Phase IV**
**EudraCT Number**	
**Coordinating Investigator**	MD, Ass. Prof. Ann-Marie Landtblom<br>Department of Neurology<br>Linköping University Hospital<br>581 85 Linköping<br>Sweden
**Sponsor**	Merck AB<br>16970 Solna<br>Sweden<br><br>**Medical Responsible**:<br>Viveka Åberg, MD<br>Medical Director<br>Phone: +46 8 562 445 13<br>Fax: +46 (0) 562 44 510
**Trial Protocol Version**	**04 December 2012 / Final Version 1.0**

**Replaces Clinical Trial Protocol Version**	**N/A**
**Current Clinical Trial Protocol Amendment**	**Not applicable**

– Confidential –

This document is the property of Merck KGaA, Darmstadt, Germany, or one of its affiliated companies. It is intended for restricted use only and may not – in full or part – be passed on, reproduced, published or used without express permission Merck KGaA, Darmstadt, Germany or its affiliate. Copyright © 2012 by Merck KGaA, Darmstadt, Germany or its affiliate. All rights reserved.

SIGNATURE PAGE

Sponsor medical representative responsible for the clinical trial:

I approve the design of the trial.

____________________________________	____________________________
Signature	*Date of Signature*

Viveka Åberg
Medical Director
Merck AB,
16903 Solna
Sweden
Phone: +46 8 562 445 13
Fax: +46 (0) 562 44 510

E-mail:viveka.aberg@merckgroup.com

Principal Investigator

Trial Title: A phase IV multicenter randomized study to assess the impact of a patient support program (MinSupport Plus) on health related quality of life (HRQoL) and adherence in patients with relapsing remitting multiple sclerosis administered Rebif® with the RebiSmart device.

EudraCT Number	2012-004887-22
Protocol Version/Date:	Dec 2012, Final Version 1.0
Centre Number	01
Principal Investigator	MD, Assistant Professor. Ann-Marie Landtblom<br>Department of Neurology, Linköping University Hospital

I, the undersigned, am responsible for the conduct of the trial at this site and affirm that:

- I understand and will conduct the trial according to the protocol, any approved protocol amendments, global guidelines and all applicable Regulatory Authority requirements and national laws.
- I will not deviate from the protocol without prior written permission from the Sponsor and prior review and written approval from the Institutional Review Board or Independent Ethics Committee, except where necessary to prevent immediate danger to the patient.

______________________________	________________________
Signature	***Date of Signature***

Ann-Marie Landtblom, MD, Ass.Prof
Departm. of Neurology
University Hospital
581 85 Linköping
Phone : +46705591670
Fax: + 46101032668
Mail: Ann-Marie.Landtblom@lio.se

Contributor and Contact Information	Contributor and Contact Information
**Anna-Maria Ek**<br>**Health Solutions**<br>Aurorum 1c<br>977 75 Luleå , Sweden<br>Phone: +46-920-407 681<br>Mobile: +46-70-275 33 07<br>Fax: +46-920-750 38<br>E-mail: Anna-Maria.Ek@healthsolutions.se	**External data management and statistical CRO(s) to be decided.**

TABLE OF CONTENTS

LIST OF ABBREVIATIONS 8

1 SYNOPSIS 10

2 SPONSOR, INVESTIGATORS AND TRIAL ADMINISTRATIVE STRUCTURE 17

3 BACKGROUND INFORMATION AND TRIAL RATIONALE 18

4 TRIAL OBJECTIVES 21

5 INVESTIGATIONAL PLAN 22
5.1 Overall Trial Design and Plan 22
5.2 Trial Population 23
5.3 Criteria for Randomization 24
5.4 Criteria for Withdrawal 24
5.4.1 Premature Discontinuation of the Trial 24
5.5 Definition of End of Trial 24

6 INVESTIGATIONAL PATIENT SUPPORT PROGRAM AND OTHER TREATMENTS IN THE TRIAL 24
6.1 Description of investigational patient support program (MInSupport PLUS) ... 24
6.2 assignment to treatment groups 27
6.3 Concomitant Medications 28
6.3.1 Required Medications 28
6.3.2 Permitted Medications 28
6.4 METHOD OF BLINDING 28

7 TRIAL PROCEDURES AND ASSESSMENTS 29
7.1 schedule of Assessments 29
7.1.1 Treatment Period 31
7.2 Assessment of efficacy 31
7.3 Assessment of Safety 33
7.3.1 Management and reporting of Adverse Events 33
7.3.2 Recording an Adverse Event 35
7.3.3 Expedited Reporting of Serious Adverse Events 35
7.3.4 Pregnancy and In Utero Exposure 37
7.3.5 Monitoring of Patients with Adverse Reactions 37

8 DATA ANALYSIS AND STATISTICS 37
8.1 Predetermination of Sample Size 37

8.2 randomization 37
8.3 Endpoints 38
8.3.1 Primary Endpoint 38
8.3.2 Secondary Endpoints 38
8.3.3 Safety Endpoints 39
8.4 further points of interest 39
8.5 analysis sets 39
8.6 Methods for Statistical Analysis 40
8.6.1 Analysis of Baseline Parameters 40
8.6.2 Analysis of Primary Endpoint 40
8.6.3 Analysis of Secondary Endpoints 40
8.6.4 Safety Analysis 40
8.6.5 Missing Data 40
8.6.6 Interim Analysis 41

9 ETHICAL AND REGULATORY ASPECTS 41
9.1 Responsibilities of the Investigator 41
9.2 Patient Information and Informed Consent 42
9.3 Patient Identification and Privacy 43
9.4 Clinical trial insurance and compensation to patients 43
9.5 Independent Ethics Committee or Institutional Review Board 43
9.6 Health Authorities 44

TRIAL MANAGEMENT 45
9.7 Case Report Form Handling 45
9.8 Source Data and Patient Files 45
9.9 Investigator Site File and Archiving 46
9.10 Monitoring, Quality Assurance and Inspection by Health Authorities 47
9.11 Changes to the Clinical Trial Protocol 47

CLINICAL TRIAL REPORT AND PUBLICATION POLICY 48
9.12 Clinical Trial Report 48
9.13 Publication 48

10 REFERENCES 49

List of Abbreviations

ADR	Adverse Drug Reaction
AE	Adverse Event
AUC	Area Under the Curve
CI	Confidence Interval
CNS	Central Nervous System
eCRF	Electronic Case Report Form
DMT	Disease Modifying Therapies
EDSS	Expanded Disability Status Score
EQ5D-5L	Euro Quality of Life Questionnaire with 5 question alternatives
FDA	Food and Drug Administration
MFIS	Modified Fatigue Impact Scale
FSS	Fatigue Severity Scale
GCP	Good Clinical Practice
GMP	Good Manufacturing Practice
HADS	Hospital Anxiety and Depression Scale
HC	Health Care
HRQoL	Health Related Quality of Life
ICH	International Conference on Harmonization
IEC	Independent Ethics Committee
IFN	Interferon
IRB	Institutional Review Board
ITT	Intention to Treat
MI	Motivating Interviewing
MS	Multiple Sclerosis
MSIS-29	Multiple Sclerosis Impact Scale-29
MSP	MinSupport Plus
PI	Principal Investigator
PP	Per Protocol
PPMS	Primary Progressive Multiple Sclerosis

QoL	Quality of Life
RRMS	Relapsing Remitting Multiple Sclerosis
SAE	Serious Adverse Event
SAP	Statistical Analysis Plan
SC	Subcutaneous
SD	Standard Deviation
SDS	Standard Deviation Score
SPC	Summary of Product Characteristics
SPMS	Secondary Progressive Multiple Sclerosis
tiw	Three Times Weekly
TNF	Tumor Necrosis Factor
WHO	World Health Organisation

1 SYNOPSIS

**Trial title**	**RebiQoL:** A phase IV multicenter randomized study to assess the impact of a patient support program (MinSupport Plus) on health related quality of life (HR**QoL**) and adherence in patients with relapsing remitting multiple sclerosis administered Rebif® with the RebiSmart device.
**Trial number**	EMR 200136-560
**EudraCT Number**	2012-004887-22
**Sponsor**	Merck AB<br>16970 Solna<br>Sweden
**Coordinating Investigator**	MD, Ass. Prof. Ann-Marie Landtblom<br>Department of Neurology<br>Linköping University Hospital<br>Sweden
**Trial center(s)/country**	This trial will be conducted in Sweden. It is estimated that 15-20 centers will participate.
**Planned trial period**	The first patient is planned to be enrolled in Dec 2012, with the last patient expected to complete the trial in Dec 2014.
**Trial objectives**	<u>Primary objective</u><br>To assess the impact of the patient support program MinSupport Plus (MSP) on HRQoL at 12 months, using the psychological scale of Multiple Sclerosis Impact Scale-29 (MSIS-29), in patients administered Rebif® with the RebiSmart device compared to patients only receiving technical support for RebiSmart.<br><u>Secondary objectives</u><br>1. To assess the impact of the patient support program MSP on:<br>• HRQoL

<table>
<tr>
<td></td>
<td>
- MSIS-29, psychological scale at 6 months
- MSIS-29, full scale at 6 and 12 months
- Euro Quality of Life Questionnaire with 5 question alternatives (Q5D-5L) at 6 and 12 months
• Adherence at 6 and 12 months
• Fatigue Severity Scale (FSS) and Fatigue Impact Scale (MFIS) at 6 and 12 months
• Depression and Anxiety (HADS) at 6 and 12 months
• Working ability at 12 months
• The number of reported adverse events at 12 months
• Patient satisfaction at 12 months
• Health care personnel satisfaction at 12 months
• Lifestyle questionnaire at 6 and 12 months
• Lifestyle goals at 12 months
• Health economy based on QoL and working ability at 12 months

2. To describe the impact of MSP in the following sub groups:
• Males and females
• Educational level
• According to severity of MS symptoms (EDSS) at baseline, patients with EDSS > 4.0 vs. $\leq$ 4.0
• According to impairment in HRQoL at baseline
• Patients with at least one lifestyle problem at baseline according to the lifestyle questionnaire

3. To describe baseline characteristics and concomitant therapies
</td>
</tr>
<tr>
<td>Trial design and plan</td>
<td>
<u>Trial Design</u>
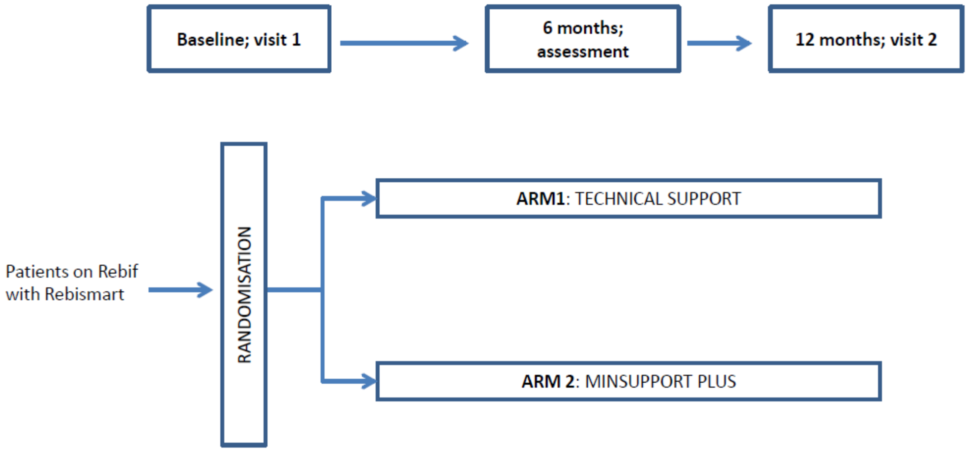

This is a randomized comparative multicenter study. The patients will be randomized to one of two arms (1:1):
</td>
</tr>
</table>

	Arm 1: Technical support for the RebiSmart device<br><br>Arm 2: Patient support program MSP<br><br><u>Visit 1</u>, baseline (Investigator visit)<br>• Patients who fulfill all eligibility criteria and sign the Informed Consent Form (ICF) will be included in the study<br>• Patient characteristics will be recorded in the eCRF (age, sex, education, marital status, time since diagnosis of RRMS, EDSS score, concomitant medication, working ability).<br>• All study questionnaires except for the lifestyle questionnaire will be handed out and completed by the patient at the clinic.<br>• After completion the patient will hand in the questionnaires to the nurse at the clinic who will check for completeness and send it to an external vendor (Health Solutions) for data entry.<br>• The Investigator/nurse at the clinic will send patient details of the included patient to Health Solutions who will randomize the patient and provide support according to randomization.<br><br><u>Six months assessment</u><br>At six months after study entry the patients in both arms will receive questionnaires from the external vendor to be filled in and sent back to the vendor.<br><br><u>Visit 2</u>, month 12 (Study nurse visit)<br>• Before the 12 months visit, the patients in both arms will receive questionnaires from the external vendor to be filled in and sent back to the vendor before the visit at the clinic.<br>• For patients randomized to the MSP arm, the web based lifestyle questionnaire is completed before the visit.<br>• Working ability will be recorded in the eCRF.<br>• Adherence data will be downloaded from the RebiSmart device.<br>• Any adverse event will be recorded in the eCRF.<br>• Health care personnel satisfaction will be recorded in the eCRF.<br><br>Study monitoring will be performed by Merck AB.
**Planned number of patients**	Assuming a group difference of 14 points (Arm 2 superior to Arm 1) and a standard deviation of 25 points on the primary endpoint a total of 104 patients are required for 80 % power and 5 % significance level with a randomization ratio of 1:1. In order to allow for a 20% drop-out rate 130 patients will be included.

<table>
<tr><td>Diagnosis and main inclusion and exclusion criteria</td><td>To be eligible for inclusion into this trial, the patients must fulfill the following criteria:
<u>Inclusion Criteria</u>
1. Male or female, aged 18 or older
2. A diagnosis of Remitting Relapsing MS (RRMS) according to the revised McDonald Criteria (2010)
3. Treatment with Rebif® 22 mcg or 44 mcg subcutaneously (SC) three times a week (tiw) in accordance to the Summary of Product Characteristics (SPC)
4. Rebif® administered by the RebiSmart™ device
5. Provided a signed ICF

<u>Exclusion Criteria</u>
1. Has received any components, except for technical support, of MinSupport Plus prior to study entry
2. Has difficulty reading and/or understanding Swedish
3. Has a mental condition rendering the patient unable to understand the nature, scope and possible consequences of the study, and/or evidence of an uncooperative attitude
4. No access to computer
5. Participation in another clinical study</td></tr>
<tr><td>Investigational Support Program</td><td><u>MinSupport Plus</u>
This program focuses on support and motivation to help patients achieve the best results with their Rebif® treatment and includes:
• Technical support for the RebiSmart device
• Personal coaching regarding treatment and understanding of the disease
• Lifestyle Guide
• Web support (personal health records)
The objective of the Lifestyle Guide is to inspire and motivate a choice of lifestyle(s) in order to improve health and wellbeing. The Lifestyle Guide provides guidance on diet, exercise, stress management, smoking, sleep and</td></tr>
</table>

	alcohol.<br>A lifestyle questionnaire will be filled out during the first week after study inclusion by all patients randomized to the MSP arm, the result will be discussed with a lifestyle counselor, support offered and goal(s) defined in line with the patient's personal need. All patients will be offered stress management regardless of the result of the lifestyle questionnaire.
**Planned treatment duration for each patient**	Planned study period for each patient is 12 months.
**Primary endpoint(s)**	Primary<br>MSIS-29 psychological scale (9 questions): Change from baseline to month 12.
**Secondary endpoint(s)**	Secondary<br>1. MSIS-29 psychological scale (9 questions): Change from baseline to month 6<br>2. MSIS-29, full scale: Change from baseline to months 6 and 12<br>3. EQ5D-5L: Change from baseline to months 6 and 12<br>4. Adherence: Proportion of patients with <10% missed injections during 6 and 12 months, measured with the software RDS 2.0<br>5. FSS and MFIS: Change from baseline to months 6 and 12<br>6. Hospital anxiety and depression scale (HADS): Change from baseline to months 6 and 12<br>7. Working ability: Change from baseline to month 12 will be assessed by recording the following information per patient:<br>o I am employed/have my own company<br>1a. My employment is based on hours/week ____<br>I work my normal working hours (Y/N)<br>1b. I now work hours/week ______<br>1c. For the rest of the time I receive (sickness, activity compensation, parental compensation)<br>o I study (indicate how much)<br>o I have full sickness (full disability pension)

	○ I have retirement<br>○ None of the above applies to me<br><br>8. Adverse Events: Proportion of patients with reported AEs at month 12<br><br>9. Patients satisfaction:<br>The following question will be answered at the 12 months visit and recorded in the eCRF: "How satisfied are you with the overall treatment and support from health care providers during the last 12 months?"<br>*Categorized as follows: Very unsatisfied / Unsatisfied / Satisfied / Very satisfied*<br>Please specify: ____________________________<br><br>10. HC Personnel satisfaction:<br>The following question will be answered once per site at the 12 months visit for the last patient at the site and recorded in the eCRF: "How satisfied are you with the overall treatment and support from health care providers during the last 12 months?"<br>*Categorized as follows: Very unsatisfied / Unsatisfied / Satisfied / Very satisfied*<br>Please specify: ____________________________<br><br>11. Lifestyle questionnaire: Change from baseline to month 6 and 12 (arm 2)<br><br>12. Lifestyle goals:<br>The following questions will be included in the questionnaires completed by the patients before the 12 months visit (arm 2):<br>- Was the goal achieved? (Y/N)<br>- If yes, better than expected or achieved as expected?<br>- If better than expected, a lot or a little better than expected?<br>- If no, a little or a lot less than expected?<br><br>13. Health economy based on QoL and working ability at 12 months
**Other assessments**	Not applicable
**Statistical Methods**	<u>Primary Endpoint Analysis</u><br><br>The primary endpoint will be analyzed using a linear mixed model with the change from baseline to month 6 and month 12 as dependent variable and group, time and baseline value of the dependent variable as fixed factors. The patient specific intercept will be included as a random factor. The difference between groups at month 6 and month 12 will be presented along with 95 % confidence intervals. The 12 month is the primary endpoint.

	The derivation from the 9 questions to the MSIS-29 psychological scale can be performed using the following equation:<br>Score (0-100)=(∑(9 questions)-9)/0.36<br><br><u>Secondary Endpoint Analysis</u><br>Numeric secondary endpoints will be analyzed using the same method as for the primary endpoint. Dichotomized endpoints (e.g. proportion of patients meeting the criteria for adherence) will be analyzed at month 6 and month 12 separately using a logistic regression. The results will be presented as odds ratios with 95 % confidence intervals.
**Estimated trial calendar**	Dec 2012 – Dec 2014

2 SPONSOR, INVESTIGATORS AND TRIAL ADMINISTRATIVE STRUCTURE

Trial Sponsor: The principal Sponsor of this Phase IV, multicenter trial will be Merck AB, Solna, Sweden.

Location(s) of the Trial: This multicenter trial will be conducted in Sweden. It is estimated that 15-20 centres will participate in the trial.

Principal Investigator: The Principal Investigator (PI) will serve as the representative(s) for Investigators at each participating trial centre for decisions and discussions regarding this trial (ICH E6 GCP, 1.19). His/her/their role will be to provide expert medical input and advice relating to the trial design and conduct, and will be responsible for the review and sign-off of the Clinical Trial Report, on behalf of all participating Investigators.

For this trial, the contact information for the Principal Investigator will be entered on page 4 of this protocol.

Third-Party Service Providers: The Sponsor will rely on an external vendor (Health Solutions) for randomizing patients and providing appropriate support (technical or patient support program) according to randomization. They will also be responsible for sending questionnaires to patients at month 6 as well as for data entry of all questionnaires (baseline, 6 and 12 months).

The Sponsor will also rely on an external vendor for data management and statistics.

Monitoring: The sponsor will be responsible for site monitoring and site closure activities. The details will be described in a Monitoring Plan.

Drug Safety Responsibilities: The Sponsor's Safety department will be responsible for overseeing the safety of Rebif ® during the conduct of this trial and will inform Health Authorities on all adverse drug reactions (ADRs) in compliance with existing regulatory requirements.

3 BACKGROUND INFORMATION AND TRIAL RATIONALE

Multiple sclerosis and the clinical variants

MS affects 2.1 million people worldwide, and in Sweden 17000. MS is the most common chronic, non traumatic neurological disorder afflicting young people during their peak productive ages. The exact cause of MS is unknown, although an autoimmune process has been implicated. Genetic susceptibility plays a role in disease initiation (1-3) but currently unidentified environmental factors are also involved (4). It is hypothesized that central nervous system (CNS) auto-reactive T-cells is stimulated in the peripheral circulation to become active and proliferate. The expression of adhesion molecules on the surface of T-cells permits adhesion to activated endothelial cells with subsequent migration of T-cells into the CNS compartment. These cells upon interacting with CNS myelin antigens again proliferate, and initiate a pro-inflammatory cascade within the brain that results in either target-directed immune damage or bystander damage. Important cellular and humoral elements are T-lymphocytes, macrophages, microglial cells, metalloproteinases and cytokines, including interferon-gamma (IFN-gamma), and tumor necrosis factor-alpha (TNF-alpha) (5-7).

Three clinical forms of MS are recognized: primary progressive MS (PPMS), secondary progressive MS (SPMS), and relapsing-remitting MS (RRMS) (8, 9). For this study we use the acronym RMS, Relapsing MS, which encompasses RRMS and relapsing SPMS patients. PPMS patients encompass about 10% of the MS population; their disease is characterized by slow and steady accumulation of neurological deficits from onset without superimposed events. A smaller percentage of patients will have a similar onset but with occasional clinical events (progressive-relapsing).

RRMS

Patients with RRMS have acute exacerbations or clinical events with subsequent variable recovery (remission). Forty to 50% of all MS patients have a relapsing-remitting (RR) course although at the onset of MS, 80 to 85% of patients will have the RR form of the disease. Most patients with Expanded Disability Status Scale (EDSS) scores <4 have the RR form of multiple sclerosis. Approximately 10% of patients have benign multiple sclerosis, a subset of RRMS, characterized by the lack of accumulation of significant residual neurological deficit over time, with EDSS scores of <3 after 10 to 15 years of disease.

Fifty percent (50%) of RRMS patients will convert to SPMS within 10 years of onset (10) with the peak time of conversion being at about eight years after the onset of the disease (1). The proportion of RRMS progressing to SPMS approaches 80% at 25 years. SPMS is characterized by the steady accumulation of significant and persistent neurological deficit with or without superimposed clinical events/relapses. Response to interferon (IFN) therapy may differ based on whether clinical events continue to occur in SPMS. The majority of patients with EDSS scores of 6.0 or higher have SPMS.

MS and Health Related Quality of Life (HRQoL)

MS can diminish HRQoL by interfering with ability to work, pursue leisure activities, and carry on usual life roles. MS is a disease that affects physically, but it can also take a toll on emotions. Many young people suffering from MS, need to quickly adapt to a new life situation, that is hard to manage on their own. This often results in anger, frustration, hopelessness and depression.

Symptoms that effect HRQoL, alongside physical symptoms, may include fatigue, stress, depression, pain, spasticity, cognitive impairment, sexual dysfunction, bowel and bladder dysfunction, vision and hearing problems, seizures, and swallowing and breathing difficulties. And that is true throughout the journey with MS, not just for those who are newly diagnosed.

Among individuals diagnosed with MS, one-third describes fatigue as the very first symptom (11) and 65% of patient with MS complain of fatigue (12). Fatigue is an overwhelming and abnormal feeling of extreme tiredness and the subjective nature of fatigue makes it hard to understand for others than those afflicted. Therefore, knowledge about the impact of fatigue on patient's daily living is extremely important. Depression and anxiety is also major complaints within the MS population. Lifetime prevalence of depression among patients with MS is about 50%, and anxiety reach prevalence of 25% (12).

The medical cost for MS patients is high. Although medical cost predominate in the earlier stages of MS, indirect costs of productivity loss are responsible for high costs later on. Disease-modifying therapies (DMTs) lessen symptoms, reduce relapses, and delay disability progression, but only modestly improve HRQoL. To help MS patients with their psychological wellbeing alongside drug treatment patient support program must be part of the general treatment of MS.

There is a high unmet need for MS patients to increase their HRQoL and in addition to drugs patients with MS need help to managing their MS. In order to increase patient's stability, adherence and motivation a patient support program, MSP has been developed for Merck AB to increase the well being in patients with MS

Treatment Adherence to MS Therapies

According to the World Health Organisation (WHO), treatment adherence is defined as both compliance (taking the medication in the correct dose and according to the schedule prescribed) and persistency (maintenance of the drug regimen over the long-term).

The degree to which patients adhere to medication regimens is difficult to assess and quantify accurately. There is no standardized tool kit for the measurement of adherence and most studies that have adherence as an outcome use indirect measures. It is clear that poor adherence is an important worldwide problem. The WHO quotes an average figure of 50% for adherence to medication for chronic diseases in developed countries, and in

developing countries the rates of adherence are even lower (13). Sub-optimal adherence is likely to adversely affect health outcomes and healthcare costs. Treatment adherence is essential to maximize treatment outcomes in MS. Previous studies have shown that patients with a gap in therapy as small as 90 days have a higher risk of severe relapse (14) compared to more adherent patients, and patients who prematurely discontinue therapy had a significantly higher EDSS score than those patients that continued treatment (15). Strategies to maintain good adherence are desperately needed to assure that MS patients derive full therapeutic benefit from their treatment. Barriers to treatment adherence have been defined in several publications (16-22) and can be categorized into those that result in premature treatment discontinuation (persistence) and those that contribute to intermittently missing doses (compliance). Patients have poor compliance due to forgetfulness, injection fatigue and injection related issues such as injection pain and injection anxiety. In many cases poor short-term compliance may be a precursor to poor persistence and premature discontinuation. For example, poor compliance shortly after initiating treatment may affect the therapeutic benefit and lead a patient to perceive a lack of treatment efficacy (16). Perceived lack of efficacy has been reported to be the cause of 30% of discontinuations from first-line treatment in MS (13). A study of patients' expectations of the effects of IFN-beta 1b treatment reported that 57% of patients had an unrealistic expectation of therapy with regard to relapse reduction, and 34% with regard to improvements in functional status (23). Similarly, injection issues, such as injection pain and injection anxiety, that precipitate missing intermittent doses (19,20) can eventually lead to premature treatment termination (16,18). Many people dislike injections, particularly when they are self-administered (17); the prevalence of injection phobia in the general population is 7–22%. Self-injection anxiety and belief that self-injection is not possible have been shown to be strong predictors of medication discontinuation at 6 months (18). The occurrence of adverse events is a major barrier to adherence to MS therapy (16). It is therefore important to avoid or manage adverse events when possible, with the aim of improving adherence. The flu-like symptoms that can accompany IFN beta therapy tend to be transient, can be mitigated by dose titration at treatment initiation, and are commonly effectively managed with concomitant administration of non-steroidal anti-inflammatory drugs (24). The incidence of injection-site reactions can be reduced with correct injection technique and by the use of injection devices (15). Therefore, any strategies that are aimed at improving treatment compliance may have a similar impact on treatment persistence and improve overall patient adherence to therapy.

The RebiSmart™ device is a CE-certified, reusable electromechanical auto-injector intended for the administration of Rebif® in multi-dose cartridges. It is a stand-alone hand-held device with an internal power supply. It is used for subcutaneous (sc) injections with single-use sterile disposable Serofine™ needles. The device is designed to inject a volume of 0.5ml per standard dose injection at a defined injection depth. The device is to be kept in a storage box and usually placed in the refrigerator after use, although it can be stored at or below 25°C for up to 14 days.

RebiSmart is the only injection device for MS patients that can measure missed injections, and is therefore a tool to measure adherence to treatment. Therefore only patients treated with the Rebismart device will be considered for inclusion into this study.

Rationale for the current study

As described above there is a high unmet need for MS patients to increase their HRQoL as well as to improve adherence to their MS treatment. The present study aim to assess the impact of the patient support program MSP on HRQoL and adherence in MS patients administered Rebif® with the RebiSmart device and thereby provide additional scientific knowledge regarding how to better treat MS patients from a holistic point of view.

This clinical trial will be conducted in compliance with the clinical trial protocol, Good Clinical Practice (ICH Topic E6, GCP) and the applicable regulatory requirements.

4 TRIAL OBJECTIVES

Primary objective

To assess the impact of the patient support program MSP on HRQoL at 12 months, using the psychological scale of MSIS-29, in patients administered Rebif with the RebiSmart device compared to patients only receiving technical support for RebiSmart.

Secondary objectives

1. To assess the impact of the patient support program MSP on:
- HRQoL
  - MSIS-29, psychological scale at 6 months
  - MSIS-29, full scale at 6 and 12 months
  - EQ5D-5L at 6 and 12 months
- Adherence at 6 and 12 months
- Fatigue (FSS, MFIS) at 6 and 12 months
- Depression and Anxiety (HADS) at 6 and 12 months
- Working ability at 12 months
- The number of reported adverse events at 12 months
- Patient satisfaction at 12 months
- Health care personnel satisfaction at 12 months
- Lifestyle goals at 12 months
- Health economy based on QoL and working ability at 12 months

2. To describe the impact of MSP in the following sub groups:
- Males and females
- Educational level
- According to severity of MS symptoms (EDSS) at baseline, patients with EDSS > 4.0 vs. ≤ 4.0

- According to impairment in HRQoL at baseline
- Patients with at least one lifestyle problem at baseline according to the lifestyle questionnaire

3. To describe baseline characteristics and concomitant therapies

5 INVESTIGATIONAL PLAN

5.1 OVERALL TRIAL DESIGN AND PLAN

Figure 1. Trial Scheme

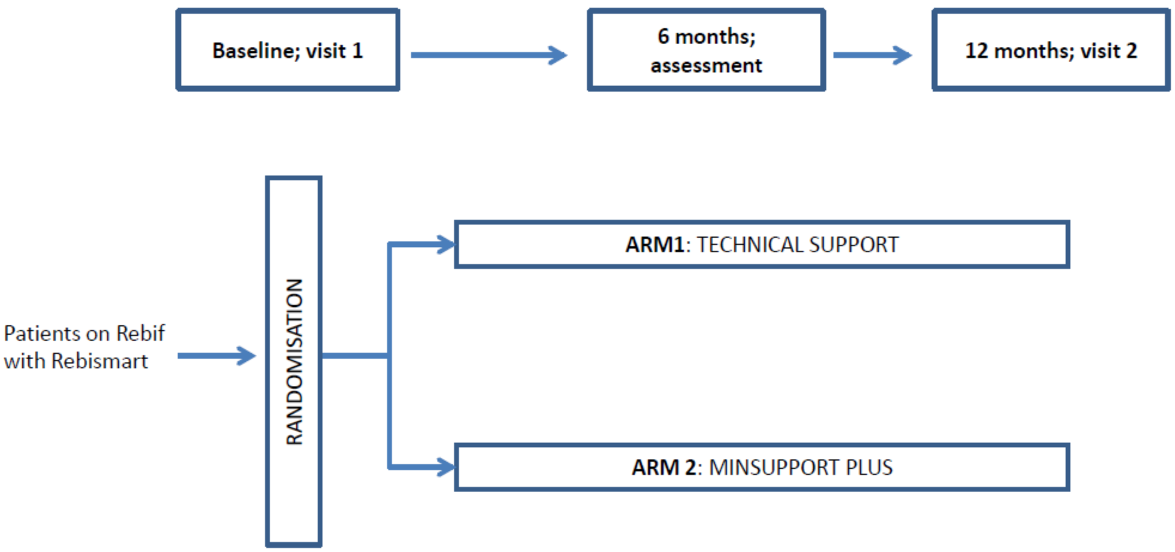

This is a randomized comparative multicenter study. The patients will be randomized to one of two arms (1:1):

Arm 1: Technical support for the RebiSmart device

Arm 2: Patient support program MSP

Study monitoring will be performed by Merck AB. Further details are provided in the monitoring plan.

The total duration of the study is planned from Dec 2012 (first patient in) to Dec 2014 (last patient out). The recruitment period will be 1 year. Each patient participates in the study for a period of 12 months.

A patient is considered to be enrolled in the study when having signed the ICF. A patient has completed the study when the investigator signs off the patient's Case Report Form (CRF) at Month 12 (visit 2) or at an early discontinuation visit.

Participating sites may start patient enrolment as soon as ethics approval is received.

5.2 TRIAL POPULATION

This trial will be conducted in Sweden at 15-20 experienced MS centres. A total of 130 MS patients will be included in the trial.

<u>Inclusion Criteria</u>

For inclusion in the trial, all of the following inclusion critera must be fulfilled:

- Male or female, aged 18 or older
- A diagnosis of RRMS according to the revised McDonald Criteria (2010)
- Treatment with Rebif® 22 mcg or 44 mcg subcutaneously (SC) three times a week (tiw) in accordance to the SPC
- Rebif® administered by the RebiSmart™ device
- Provided a signed ICF

<u>Exclusion Criteria</u>

Patients are not eligible for this trial if they fulfill any of the following exclusion criteria:

- Has received any components, except for technical support, of MSP prior to study entry.
- Has difficulty reading and/or understanding Swedish
- Has a mental condition rendering the patient unable to understand the nature, scope and possible consequences of the study, and/or evidence of an uncooperative attitude.
- No access to computer

- Participation in another clinical study

5.3 CRITERIA FOR RANDOMIZATION

Once a patient has fulfilled all the inclusion and exclusion criteria and signed the ICF, the patient is included in the study. The investigator/nurse will send patient details of the included patient to Health Solutions who will randomize the patient and provide support according to randomization. The randomization will be stratified for sex and severity of disease.

5.4 CRITERIA FOR WITHDRAWAL

Patients will be informed that they have the right to withdraw from the trial at any time, without prejudice to their medical care, and that they are not obliged to state their reason(s). Any withdrawal must be fully documented in the eCRF and source documents, and should be followed up by the Investigator.

The Investigator may withdraw a patient at any time if this is considered to be in the patient's best interest.

If the study patient support program is prematurely discontinued, the primary reasons for discontinuation must be recorded in the appropriate section of the eCRF.

The Investigator/nurse will arrange for appropriate follow-up according to visit 2 for patients withdrawing from the study.

5.4.1 Premature Discontinuation of the Trial

In case of study discontinuation, all national regulatory procedures will be followed.

5.5 DEFINITION OF END OF TRIAL

For administrative and safety reporting purposes, the end of the trial will be defined as the date of the final clinical database lock. This provides for a single and conservative definition across all trial centres.

6 INVESTIGATIONAL PATIENT SUPPORT PROGRAM AND OTHER TREATMENTS IN THE TRIAL

6.1 DESCRIPTION OF INVESTIGATIONAL PATIENT SUPPORT PROGRAM (MINSUPPORT PLUS)

MSP is a service from Merck AB which includes:

- Technical support for the RebiSmart device
- Personal coaching regarding treatment and understanding of the disease
- Lifestyle Guide
- Web support (personal health records)

The patient is assigned a personal nurse/coach and a personal health counselor/advisor. The first two services described below are performed by the personal nurse and the third service by the personal health counselor.

1. Technical support

The personal nurse provides technical support for the RebiSmart device.

2. Treatment and understanding of the disease

The personal nurse supports and guides the patient in adherence to the prescribed Rebif® therapy, provides disease information and motivating support for the treatment of MS. The nurse checks the patient's understanding of the doctor's prescription, the administration of the medicine and makes continuous evaluation of patient’s status, issues, and problems with regard to treatment using a set of questions in the patient's health record (“Hälsojournal”).

This support is managed by;

- A set of telephone contacts with the patient; 1 registration call followed by 6 motivational calls during 12 months. The calls to the patients are booked 1-7 days in advance
- The telephone calls are supported by customized mailings and mobile text messaging to the patients

4. Lifestyle Guide

This support is provided by a personal health counselor. The purpose of the Lifestyle Guide is to inspire and motivate a choice of lifestyle(s) in order to improve health and wellbeing. The Lifestyle Guide is based on The Swedish National Board of Health and Welfare’s National Guidelines for Methods of Preventing Disease. These guidelines provide recommendations for methods of preventing disease by supporting patients in their efforts to change an unhealthy lifestyle habit. The Lifestyle Guide offers guidance in six areas; diet, exercise, stress management, smoking, sleep and alcohol. Counselling is individual, which means that the patient agrees with the lifestyle counsellor which areas to focus on and in which order. Most people who start with lifestyle changes will end in 6-10 weeks (30). As our intention is to achieve long term change, the program runs during 12 months.

The Lifestyle Guide is managed by;

- An Introductory questionnaire which concerns different areas that affects health. The patient may also, in the questionnaire, indicate their willingness for change in each area (judging on a scale of 1 to 10).
- 6 motivating conversation with the personal health counselor.
  - The first conversation includes an initial analysis of individual needs and goal formulation based on the completed questionnaire. All patients will be offered stress management regardless of the result of the questionnaire as well as other support based on the analysis of individual needs.
  - The patients will formulate their own goals. The patient's goals and goal fulfillment are important in the process of change.
  - Five follow-up and motivating conversations will be distributed over a year.

3. Webb Support

The patient has access to two web-based personal health records, which can be adapted individually for optimal support:

- One managed by the personal nurse for coaching of treatment and understanding of the disease documented in a Health Record ("Hälsojournal" in Swedish)
- One managed by the personal health counsellor for coaching of life style documented in a Health Diary ("Hälsodagbok" in Swedish)

The information reported by the patient is used to provide individual feedback and to determine the content of the coaching conversations.

Health Solutions AB

MSP will be provided by Health Solutions AB, a registered healthcare company active in two areas; diagnosis-specific patient support and patient support for lifestyle changes. Health Solution AB is located in Luleå, Sweden.

The coaching includes several different communication alternatives; telephone, e-mail, outbound text messaging, online chats, as well as the technical platform RealQ supporting the personal health records. The patient can contact the personal coaches via telephone, instant messaging or e-mail according to his or her own needs. All calls are documented in the health records. In addition, all incoming mails to the personal nurse are documented in the patient's health record ("Hälsojournal" in Swedish).

The calls are performed according to the Motivating Interviewing (MI) method and are individually adjusted for the patients' need. The calls are coordinated with mail and mobile text messaging.

MI refers to a counselling approach in part developed by clinical psychologists Professor William R Miller, Ph.D. and Professor Stephen Rollnick, Ph.D. It is a client-centered, semi-directive method of engaging intrinsic motivation to change behaviour by developing discrepancy and exploring and resolving ambivalence within the client (31). This interview method aims to increase the individual's inner motivation for change and to strengthen his or her commitment to implementing change.

Schematic overview of coaching

The timing of the respective coaching of the personal nurse and personal health counselor is presented in the table below.

Time for call from patient inclusion		MinSupport	Lifestyle Guide
**Month**	**Week**	**Personal Nurse**	**Personal Health Counselor**
1	1	Registration call	Call 1
	2		Call 2
	3	Call 1	
2	2		Call 3
	3	Call 2	
3	2		Call 4
	3	Call 3	
5	2		Call 5
	3	Call 4	
8	3	Call 5	
9	2		Call 6
11	3	Call 6	
**No of calls**		**7**	**6**

6.2 ASSIGNMENT TO TREATMENT GROUPS

The patient will be randomized to one of two study arms (1:1):

Arm 1: Technical support for the RebiSmart device

Arm 2: Patient support program MSP

The randomization will be stratified for sex and severity of disease.

6.3 CONCOMITANT MEDICATIONS

Concomitant medication is defined as all other pharmaceutical therapy administered during the study.

6.3.1 Required Medications

Treatment with Rebif® 22/44 mcg as administered by the RebiSmart device and in accordance to the SPC (see Appendix I). The decision to prescribe Rebif® must have been made independently of and prior to considering inclusion into this study.

Patients discontinuing treatment with Rebif® due to any reason during the study period should continue assessments, participation in the support program and perform final visit. The reason for discontinuing Rebif® must be recorded in the eCRF. These patients will be included in the Intention-To-Treat (ITT) analysis and be excluded from the per protocol (PP) analysis.

6.3.2 Permitted Medications

Any medication considered necessary for the patient's welfare, may be given at the discretion of the Investigator.

6.4 METHOD OF BLINDING

At visit 1, the patients will complete all questionnaires (except for the Lifestyle questionnaire), i.e. before allocation to study arm which will be performed by Health Solutions during the first week. The questionnaires will be sent for data entry to a unit within Health Solutions which is separate to the unit providing the patient support. At 6 and 12 months the questionnaires will be sent out directly to the patients for completion and the patients will return completed questionnaires to the separate unit at Health Solutions for data entry. The entered data will be transferred to an external vendor for data management and statistics. Thus, the Investigator/Study nurse will be blinded with respect to study arms unless the patient actively reveals it to the Investigator/Study nurse. In the patient information the patients will be asked not to reveal which study arm they are allocated to.

7 TRIAL PROCEDURES AND ASSESSMENTS

7.1 SCHEDULE OF ASSESSMENTS

Prior to performing any trial assessments that are not part of the patients' routine medical care, the Investigator will ensure that the patient has provided written informed consent. Date of informed consent signature must be recorded in the eCRF.

Visit 1, baseline (Investigator visit)

- Patients who fulfill all eligibility criteria and sign the ICF will be included in the study

- The following patient characteristics are recorded in the eCRF:
    - Age (years)
    - Sex
    - Education
    - Marital status and housing situation
    - Medical history
    - Concomitant medication
    - Working ability

- Questionnaires:
    - All study questionnaires except for the lifestyle questionnaire will be handed out and completed by the patient at the clinic at the time of the baseline visit.
    - After completion the patient will hand in the questionnaire to the nurse at the clinic who will check for completeness and send it to an external vendor for data entry.
    - Lifestyle questionnaire: For patients randomized to the MSP arm, a web based form will be filled out during the first study week (Arm 2).

- Randomization: The Investigator/nurse at the clinic will send patient details of the included patient to Health Solutions who will randomize the patient and provide support to the patient according to randomization.

- Lifestyle goal(s): Personal lifestyle goals will be defined and recorded in the health diary during the first study week (Arm 2).

Six months assessment

- At six months after study entry the patients in both arms will receive questionnaires from the external vendor (Health Solutions) to be filled in and sent back to the vendor.
- For patients randomized to the MSP arm the web based lifestyle questionnaire is completed.

Visit 2, month 12 (Study nurse visit)

- Before the 12 months visit, the patients in both arms will receive questionnaires from the external vendor to be filled in and sent back to the vendor before the visit at the clinic.
- For patients randomized to the MSP arm, the web based lifestyle questionnaire is completed before the visit.
- Working ability will be recorded in the eCRF.
- Adherence data will be downloaded from the RebiSmart device.
- Any adverse event will be recorded in the eCRF.
- Health care personnel satisfaction will be recorded in the eCRF once per site, at the 12 months visit for the last patient at the site.

7.1.1 Treatment Period

The study period for each patient is 12 months. Study visits and schedule of assessments are summarised in the table below.

VISIT	Baseline **Visit 1** (Investigator)	6 month **Assessment**	12 month **Visit 2** (Study nurse)
**Clinical assessments**			
Informed consent signature	X		
Patient characteristics[1]	X		
Working ability	X		X
Concomitant Medication	X		X
Questionnaires[2]	X	X	X
Randomization	X[3]		
Lifestyle Questionnaire[4]	X	X	X
Lifestyle Goals[4]	X		X
Patient satisfaction			X
HC Personnel satisfaction[5]			X
Adherence to treatment			X
Adverse Events			X
End of Trial Information			X

1. Age, sex, education, marital status, housing situation and medical history
2. MSIS-29, EQ5D-5L, HADS, FSS, MFIS, and MUSIQoL selected questions
3. Performed by Health Solutions during first study week
4. For patients randomized to the MSP arm
5. Once per site, at the 12 months visit for the last patient at site

7.2 ASSESSMENT OF EFFICACY

<u>Primary endpoint</u>

MSIS-29 psychological scale (9 questions): Change from baseline to month 12.

Secondary endpoints

- MSIS-29 psychological scale (9 questions): Change from baseline to month 6
- MSIS-29, full scale: Change from baseline to months 6 and 12
- EQ5D-5L: Change from baseline to months 6 and 12
- Adherence: Proportion of patients with <10% missed injections during 6 and 12 months, measured with the software RDS 2.0
- FSS and MFIS: Change from baseline to months 6 and 12
- HADS: Change from baseline to months 6 and 12
- Working ability: Change from baseline to month 12 will be assessed by recording the following information per patient:
  - I am employed/have my own company
    1a. My employment is based on hours/week ____
    I work my normal working hours (Y/N)
    1b. I now work hours/week ______
    1c. For the rest of the time I receive (sickness, activity compensation, parental compensation)
  - I study (indicate how much)
  - I have full sickness (full disability pension)
  - I have retirement
  - None of the above applies to me
- Adverse events: Proportion of patients with reported AEs at month 12
- Patients satisfaction:
  The following question will be answered at the 12 months visit and recorded in the eCRF: "How satisfied are you with the overall treatment and support from health care providers during the last 12 months?"
  Categorized as follows: Very unsatisfied / Unsatisfied / Satisfied / Very satisfied
  Please specify: ____________________________
- HC Personnel satisfaction:
  The following question will be answered once per site at the 12 months visit for the last patient at the site and recorded in the eCRF: "How satisfied are you with the overall treatment and support from health care providers during the last 12 months?"

Categorized as follows: Very unsatisfied / Unsatisfied / Satisfied / Very satisfied
Please specify: ____________________________

- Lifestyle questionnaire: Change from baseline to month 6 and 12 (arm 2)

- Lifestyle goals:
  The following questions will be included in the questionnaires completed by the patients before the 12 months visit (arm 2):
  - Was the goal achieved? (Y/N)
  - If yes, better than expected or achieved as expected?
  - If better than expected, a lot or a little better than expected?
  - If no, a little or a lot less than expected?

- Health economy based on QoL and working ability at 12 months

- Questionnaires:

  - **MSIS-29** (Appendix II), a validated MS specific questionnaire consisting of 29 questions of which 20 address the physical impact component and nine assess the psychological impact. A combined score can be generated, or both components can be reported separately (25, 26).
  - **EQ5D-5L** (Appendix III), a standardized questionnaire to measure health and HRQoL, which can be used regardless of the patient's illness or treatment (27).
  - **HADS** (Appendix IV), to measure depression and anxiety in patients. The scale is limited to 14 questions, a practical tool for identifying and quantifying the two most common forms psychological disturbances in medical patients (28).
  - **FSS** (Appendix V), is a method of evaluating fatigue in multiple sclerosis and is designed to differentiate fatigue from clinical depression, since both share some of the same symptoms. FSS consists of answering 9 questions that require the subject to rate his or her own level of fatigue.
  - **MFIS** (Appendix VI) is a modified version of Fatigue Impact Scale (FIS) (29), based on items derived from interviews with MS patients concerning how fatigue impacts their lives. The instrument provides an assessment of the effects of fatigue in terms of physical, cognitive and psychosocial functioning. MFIS consists of 21 items.
  - **MUSIQoL** (Appendix VII), selected questions regarding memory and sex life.

7.3 ASSESSMENT OF SAFETY

7.3.1 Management and reporting of Adverse Events

Adverse events will be documented during the whole observational period, starting at the baseline visit, after signing of the informed consent form by the patient (both conditions must be met). Recording of adverse events stops when the Investigator signs off the eCRF at Month 12 or during an early discontinuation visit.

Definitions

Adverse Event:
An adverse event (AE) is any untoward medical occurrence in a patient or a clinical trial subject, administered a pharmaceutical product, which does not necessarily have a causal relationship with this treatment. An AE can therefore be any unfavorable and unintended sign (including an abnormal laboratory finding), symptom, or disease temporally associated with the use of a medicinal product, whether or not considered related to the medicinal product.

In cases of surgical or diagnostic procedures, the condition/illness leading to such a procedure is considered as the AE rather than the procedure itself.

In case of a fatality, the cause of death is considered as the AE, and the death is considered as its OUTCOME.

The Investigator is required to grade the severity/intensity of each adverse event.

Adverse Drug Reaction (ADR):
A response to a medicinal product which is noxious and unintended. Response in this context means that a causal relationship between a medicinal product and an adverse event is at least a reasonable possibility. Adverse reactions may arise from use of the product within or outside the terms of the marketing authorisation or from occupational exposure. Conditions of use outside the marketing authorisation include off-label use, overdose, misuse, abuse and medication errors.

Serious Adverse Event:

- Results in death.
- Is life-threatening at time of event.
- Requires in-patient hospitalization or prolongation of existing hospitalization.
- Results in persistent or significant disability/incapacity.
- Is a congenital anomaly/birth defect.
- Is otherwise considered as medically important.

Severity of an Adverse Event:

Investigators must assess the severity/intensity of Adverse Events according to the Qualitative Toxicity Scale, as follows:

Mild: The patient is aware of the event or symptom, but the event of symptom is easily tolerated.

Moderate: The patient experiences sufficient discomfort to interfere with or reduce his or her usual level of activity.

Severe: Significant impairment of functioning: the patient is unable to carry out usual activities.

Investigators must also systematically assess the causal relationship of AEs to Rebif using the following definitions. Decisive factors for the assessment of causal relationship of an AE to Rebif include, but may not be limited to, temporal relationship between the AE and Rebif, known side effects Rebif, medical history, concomitant medication, course of the underlying disease, trial procedures

- Not related: Not suspected to be reasonably related to Rebif. AE could not medically (pharmacologically/clinically) be attributed to Rebif under study in this clinical trial protocol. A reasonable alternative explanation must be available.
- Related: Suspected to be reasonably related to Rebif. AE could medically (pharmacologically/clinically) be attributed to Rebif under study in this clinical trial protocol.

7.3.2 Recording an Adverse Event

Each adverse event occurring during the study, whether serious or not, and whether common during MS treatment or not (such as flu-like symptoms), must be recorded in the eCRF, including its description, seriousness criteria, severity, duration (onset and resolution dates/times), relationship with the study treatment, actions taken (reduction/discontinuation of medication; other treatment or medication; other action) and outcome.

In addition, for all Serious Adverse Events, additional information must be completed and sent to the Sponsor (see next section).

7.3.3 Expedited Reporting of Adverse Events

The Investigator must notify the Sponsor WITHIN 24 HOURS of awareness of a new Adverse Event or of new information on a previously reported Adverse Event (= "follow-up").

To do so, the Investigator must complete the adverse event section of the eCRF.

If the event is non-serious an electronic notification will be sent to the Sponsor's Local Drug Safety department automatically, who will extract relevant data for reporting.

If the event is serious, the Investigator must also complete the Sponsor Serious Adverse Event Report Form/Fax template located in the eCRF, follow the eCRF's completion instructions, and fax it directly to the Sponsor's Local Drug Safety department. Reporting by e-mail is also possible.

Fax: +46 8 562 445 10

Email address: drugsafety.sweden@merckgroup.com

For any new Adverse Event, the following minimum information is required as initial notification:

- Investigator name and contact details;
- Patient identification (study number, patient number, sex, age, …);
- Merck Drug(s);
- Description of Adverse Event.
- Assessment of the causal relationship of AEs to Rebif

Note: the Fax template found in the eCRF is a standard form for reporting Serious Adverse Events. Its fields will not fully reflect all study designs and situations. Information should be provided based on what can be found in the eCRF (i.e., the RebiQoL protocol number + site number + patient number in the "patient ID" field, even if the number format does not perfectly match), as well as anything deemed important, rather than too strictly trying to follow the format of the reporting form.

When information is communicated via telephone, a written report must be sent immediately thereafter by fax or e-mail, again using the Fax template found in the eCRF. If some data are missing, the form has to be completed with the available data and a follow-up report must be sent as soon as possible.

The Investigator/Reporter must respond to any request for follow-up information or questions the Sponsor may have regarding the AE within the same timelines as for initial reports.

Serious Adverse Drug Reactions will be expedited by the Sponsor to Competent Health Authorities according to local regulations, which usually require a maximum period of 15 days. Non-serious Adverse Drug Reactions will be expedited by the Sponsor to Competent Authorities according to local regulations, which usually require a maximum period of 90 days.

Further to reporting Adverse Events to the Sponsor, the Investigator will comply with any local Pharmacovigilance requirements to report Adverse Reactions or SAEs to national Pharmacovigilance Systems (if applicable).

7.3.4 Pregnancy and In Utero Exposure

While not considered Adverse Events, all pregnancies occurring from the date of Informed Consent signature until four (4) weeks after the end of the observational study must be reported to the Sponsor's Global Drug Safety Officer, using the contact details found in the previous section. Active follow-up of all pregnancies should be performed, even if the patient was withdrawn from the study, as is usual practice. Investigators must actively follow-up, document and report to the Sponsor on the outcome of all these pregnancies. The Sponsor's Local Drug Safety unit might provide a special reporting form in case of a pregnancy.

7.3.5 Monitoring of Patients with Adverse Drug Reactions

Any AE that occurs during the course of a clinical trial and is considered related to Rebif® , and thereby classified as an Adverse Drug Reaction, must be monitored and followed up by the Investigator until stabilization or until the outcome is known, unless the patient is documented as "lost to follow-up". This monitoring will be discontinued for non-serious ADRs at the time of database lock. Reasonable attempts to obtain this information must be made and documented. It is also the responsibility of the Investigator to ensure that any necessary additional therapeutic measures and follow-up procedures are performed.

8 Data Analysis and Statistics

Statistical analysis will be performed by an external vendor. A detailed description of all analysis will be presented in a separate Statistical Analysis Plan.

8.1 PREDETERMINATION OF SAMPLE SIZE

Assuming a group difference of 14 points (Arm 2 superior to Arm 1) and a standard deviation of 25 points on the primary endpoint a total of 104 patients are required for 80 % power and 5 % significance level with a randomization ratio of 1:1. In order to allow for a 20% drop-out rate 130 patients will be included.

8.2 RANDOMIZATION

The patients will be randomized to one of two arms (1:1):

Arm 1: Technical support for the RebiSmart device

Arm 2: Patient support program MSP

The randomization will be stratified for sex (M, F) and severity of disease (EDSS > 4.0 vs. ≤ 4.0 at baseline.

At visit 1 the Investigator/nurse at the clinic will send patient details of the included patient to Health Solutions who will randomize the patient based on 4 separate listings provided by the statistician;

List 1: Female and mild disease
List 2: Female and severe disease
List 3: Male and mild disease
List 4: Male and severe disease

8.3 ENDPOINTS

8.3.1 Primary Endpoint

MSIS-29 psychological scale (9 questions): Change from baseline to month 12.

8.3.2 Secondary Endpoints

- MSIS-29 psychological scale (9 questions): Change from baseline to month 6
- MSIS-29, full scale: Change from baseline to months 6 and 12
- EQ5D-5L: Change from baseline to months 6 and 12
- Adherence: Proportion of patients with <10% missed injections during 6 and 12 months, measured with the software RDS 2.0
- FSS and MFIS: Change from baseline to months 6 and 12
- HADS: Change from baseline to months 6 and 12
- Working ability: Change from baseline to month 12 will be assessed by recording the following information per patient:
    - I am employed/have my own company
      1a. My employment is based on hours/week ____
      I work my normal working hours (Y/N)
      1b. I now work hours/week ______
      1c. For the rest of the time I receive (sickness, activity compensation, parental compensation)
    - I study (indicate how much)
    - I have full sickness (full disability pension)
    - I have retirement
    - None of the above applies to me
- AEs: Proportion of patients with reported AEs at month 12

- Patients satisfaction:
  The following question will be answered at the 12 months visit and recorded in the eCRF: “How satisfied are you with the overall treatment and support from health care providers during the last 12 months?"
  Categorized as follows: Very unsatisfied / Unsatisfied / Satisfied / Very satisfied
  Please specify: ______________________________

- HC Personnel satisfaction:
  The following question will be answered once per site at the 12 months visit for the last patient at the site and recorded in the eCRF: “How satisfied are you with the overall treatment and support from health care providers during the last 12 months?"
  Categorized as follows: Very unsatisfied / Unsatisfied / Satisfied / Very satisfied
  Please specify: ______________________________

- Lifestyle questionnaire: Change from baseline to month 6 and 12 (arm 2)

- Lifestyle goals:
  The following questions will be included in the questionnaires completed by the patients before the 12 months visit (arm 2):
  - Was the goal achieved? (Y/N)
  - If yes, better than expected or achieved as expected?
  - If better than expected, a lot or a little better than expected?
  - If no, a little or a lot less than expected?

- Health economy based on QoL and working ability at 12 months

8.3.3 Safety Endpoints

Any adverse event will be recorded by the nurse at visit 2 (12 months). The proportion of patients with adverse events reported will be compared between the two study arms.

8.4 FURTHER POINTS OF INTEREST

8.5 ANALYSIS SETS

The Intent-to-Treat (ITT) population is defined as all patients randomized into the trial and have completed at least one post-baseline assessment of the questionnaires.

The PP population is defined as all patients randomized into the trial and who are protocol compliant (compliance covers such considerations as exposure to treatment, availability of measurements and absence of major protocol violations).

The Safety Population is defined as all patients randomized

Full descriptions for the criteria for each population (e.g. definitions of major deviations) will be presented in the Statistical Analysis Plan.

8.6 METHODS FOR STATISTICAL ANALYSIS

8.6.1 Analysis of Baseline Parameters

Descriptive summary statistics (n, mean, standard deviation (SD), median, minimum, maximum) or frequency counts of demographic and baseline data (age, sex, education, marital status, time since diagnosis of RRMS, severity of disease (EDSS), concomitant medications, HRQoL, depression/anxiety and fatigue scores) will be presented by treatment group and overall for the ITT and PP populations (no statistical test will be performed).

8.6.2 Analysis of Primary Endpoint

The primary endpoint will be analyzed using a linear mixed model with the change from baseline to month 6 and month 12 as dependent variable and group, time and baseline value of the dependent variable as fixed factors. The patient specific intercept will be included as a random factor. The difference between groups at month 6 and month 12 will be presented along with 95 % confidence intervals. The 12 month is the primary endpoint.

The derivation from the 9 questions to the MSIS-29 psychological scale can be performed using the following equation:

Score (0-100)=(∑(9 questions)-9)/0.36

8.6.3 Analysis of Secondary Endpoints

Numeric secondary endpoints will be analyzed using the same method as for the primary endpoint. Dichotomized endpoints (e.g. proportion of patients meeting the criteria for adherence) will be analyzed at month 6 and month 12 separately using a logistic regression. The results will be presented as odds ratios with 95 % confidence intervals.

8.6.4 Safety Analysis

Data on AEs will be collected at scheduled and unscheduled visits, based on information spontaneously provided by the patient.

Summary tables of incidence and observed number of treatment emergent AEs will be presented by study arm and sorted by system organ class (SOC) and by preferred term (PT). Summary tables will be presented for (1) all AEs, (2) all AEs by severity and (3) all serious AEs.

8.6.5 Missing Data

No data will be imputed; only reported values will be used for the purpose of this analysis.

8.6.6 Interim Analysis

No interim analysis is planned for this trial.

9 ETHICAL AND REGULATORY ASPECTS

Ethical Background; This interventional study focuses on the quality of life of patients using the patient support program compared to those not receiving this support. The highest possible standards of professional conduct and confidentiality will always be maintained and legislation on data protection followed. The local representative of the Sponsor and Investigators will follow relevant national legislation, which include submitting the study to an Independent Ethics Committee (IEC).

Conduct of the Study; This study is to be performed in accordance with the protocol, the Declaration of Helsinki, and all applicable national regulatory requirements.

Independent Ethics Committee Requirements; Before initiation of the study at a given centre, written approval of the protocol, ICF, Patient Information and any other information presented to potential patients must be obtained from the appropriate IEC. Where required by local regulations, the local representatives of the Sponsor are responsible for obtaining IEC approval of the study.

Regulatory Authority Approval/Notification; As the intervention in this study is a patient support program regulatory authority approval is not needed, which has been confirmed in writing by the Medical Product Agency.

InformedConsent: The informed consent process will be in accordance with the Declaration of Helsinki in the version of Seoul 2008 and local regulatory requirements. Every potentially eligible patient will be informed of the study objectives and overall requirements. Before starting any documentation of patient data, the treating physician will explain the study fully to the patient using the Patient Information, which will be translated in the local language. Then the patient will be given the opportunity to ask additional questions. If the patient is willing to take part in the study, he/she will be asked to give written informed consent after being given sufficient time to consider participation and the opportunity to ask for further information. The ICF will be signed and personally dated by the patient, as well as by the Principal Investigator.

9.1 RESPONSIBILITIES OF THE INVESTIGATOR

The Investigator is responsible for the conduct of the trial at his/her site. He/she will ensure that the trial is performed in accordance with the clinical trial protocol and with the ethical principles that have their origin in the Declaration of Helsinki, as well as with the ICH Note for Guidance on Good Clinical Practice (ICH Topic E6, 1996) and applicable regulatory requirements. In particular, the Investigator must ensure that only patients who have given their informed consent are included into the trial.

In 1998, the US Food and Drug Administration (FDA) introduced a regulation (21 CFR, Part 54) entitled "Financial Disclosure by Clinical Investigators". For trials conducted in

any country that could result in a product submission to the FDA for marketing approval and could contribute significantly to the demonstration of efficacy and safety of Rebif® (named “covered trials” by the FDA), the Investigator and all sub-Investigators are obliged to disclose any financial interest which they, their spouses or their dependent children may have in the Sponsor or the Sponsor’s product under study. This information is required during the trial and for 12 months following completion of the trial.

9.2 PATIENT INFORMATION AND INFORMED CONSENT

An unconditional prerequisite for a patient’s participation in the trial is his/her written informed consent. The patient’s written informed consent to participate in the trial must be given before any trial-related activities are carried out.

Adequate information must therefore be given to the patient by the Investigator before informed consent is obtained (a person designated by the Investigator may give the information, if permitted by local regulations). A patient information sheet in the local language and prepared in accordance with the Note for Guidance on Good Clinical Practice (ICH Topic E6, 1996) will be provided by the Sponsor for the purpose of obtaining informed consent. In addition to providing this written information to a potential patient, the Investigator or his/her designate will inform the patient verbally of all pertinent aspects of the trial. The language used in doing so must be chosen so that the information can be fully and readily understood by lay persons.

Depending on national regulations, a person other than the Investigator may inform the patient and sign the ICF, as above.

Where the information is provided by the Investigator, the ICF must be signed and personally dated by the patient and the Investigator.

The signed and dated declaration of informed consent will remain at the Investigator’s site, and must be safely archived by the Investigator so that the forms can be retrieved at any time for monitoring, auditing and inspection purposes. A copy of the signed and dated information and ICF should be provided to the patient prior to participation.

Whenever important new information becomes available that may be relevant to the patient’s consent, the written patient information sheet and any other written information provided to patients will be revised by the Sponsor and be submitted again to the IEC/IRB for review and favorable opinion. The agreed, revised information will be provided to each patient in the trial for signing and dating. The Investigator will explain the changes to the previous version.

9.3 PATIENT IDENTIFICATION AND PRIVACY

A unique patient number will be assigned to each patient by the Investigator at inclusion, immediately after informed consent has been obtained. This number will serve as the patient's identifier in the trial as well as in the clinical trial database.

The patient's data collected in the trial eCRF will be stored under this number. Only the Investigator will be able to link the patient's eCRF trial data to the patient via an identification list kept at the site. The patient's original medical data that are reviewed at the site during source data verification by the Monitor, audits and Health Authority inspections will be kept strictly confidential. Health Solutions will receive contact information from the patients in order to be able to perform the coaching. At the end of the study all questionnaires (original data) will be sent back to the investigator for filing.

Data protection and privacy regulations will be observed in capturing, forwarding, processing, and storing patient data. Patients will be informed accordingly, and will be requested to give their consent on data handling procedures in accordance with national regulations.

9.4 CLINICAL TRIAL INSURANCE AND COMPENSATION TO PATIENTS

Insurance is covered by the state insurances.

9.5 INDEPENDENT ETHICS COMMITTEE OR INSTITUTIONAL REVIEW BOARD

Prior to commencement of the trial at a given site, the protocol will be submitted together with its associated documents (patient information and informed consent, contracts with site, CV, resource certificate, and patient questionnaires) the responsible Independent Ethics Committee (IEC)/Institutional Review Board (IRB) for its favorable opinion/approval. The written favorable opinion/approval of the IEC/IRB will be filed by the Investigator and a copy will be filed in the Trial Master File at the Sponsor.

The trial must not start at a site before the Sponsor has obtained written confirmation of favorable opinion/approval from the concerned IEC/IRB. The IEC/IRB will be asked to provide documentation of the date of the meeting at which the favorable opinion/approval was given, and of the members and voting members present at the meeting. Written evidence of favorable opinion/approval that clearly identifies the trial, the protocol version, and the Patient Information and Consent Form version reviewed should be provided. Where possible, copies of the meeting minutes should be obtained.

Amendments to the protocol will also be submitted to the concerned IEC/IRB, before implementation in case of substantial changes. Relevant safety information will be

submitted to the IEC/IRB during the course of the trial in accordance with national regulations and requirements.

9.6 HEALTH AUTHORITIES

Not applicable.

TRIAL MANAGEMENT

9.7 CASE REPORT FORM HANDLING

The main purpose of the eCRF is to obtain those data required by the study protocol in a complete, accurate, legible and timely fashion. The data in the eCRF should be consistent with the relevant source documents.

The data collected in the course of this trial must be documented in the eCRFs and/or the Adverse Event Safety Report Form (Clinical Trials), and will be collected by the sponsor. They will then be processed, evaluated, and stored in anonymous form in accordance with applicable data protection regulations.

The Investigator must ensure that the eCRFs and any other associated documents contain no mention of any patient names.

The eCRFs must be filled in completely and legibly, using either black or blue ballpoint pen suitable for use on official documents. Any necessary amendments and corrections must be undertaken and countersigned by the Investigator, stating the date of the amendment/correction. Errors must remain legible, and may not be deleted with correction aids (e.g., Tipp-Ex®). The Investigator must state his/her reasons for the correction of important data.

In the case of missing data or remarks, the entry spaces provided in the eCRF should be cancelled out to avoid unnecessary follow-up inquiries.

The eCRFs are essential trial documents and must be suitable for authority inspections and submission to authorities.

The data will be entered into a validated database. An external vendor will be responsible for data processing, in accordance with the Sponsor's data management procedures. Database lock will occur once quality control procedure, and quality assurance procedures (if applicable) have been completed. PDF files of the eCRFs will be provided to the Investigators at the completion of the trial.

9.8 SOURCE DATA AND PATIENT FILES

The Investigator must keep a patient file (medical file, original medical records) on paper or electronically for every patient included in the trial. This file will contain the available demographic and medical information for the patient, and should be as complete as possible. In particular, the following data should be available in this file:

- Patient's full name,
- Date of birth,
- Sex,
- Medical history and concomitant diseases,

- Concomitant therapies (including changes during the trial),
- Trial identification
- Date of patient's inclusion into the trial (i.e. date of giving informed consent),
- Patient number in the trial,
- Dates of the patient's visits to the site,
- All adverse events observed in the patient,
- Date of patient's end of trial, and
- Date of and reason for early withdrawal of the patient from the trial or from Rebif®, if applicable.

It must be possible to identify each patient by using this patient file.

Additionally, any other documents containing source data must be filed. This includes all questionnaires. Documents must bear at least the patient number and the date when the procedure was performed. Information should be printed by the instrument used to perform the assessment or measurement, if possible. Information that cannot be printed by an automated instrument will be entered manually. Medical evaluation of such records should be documented as necessary and the documentation signed and dated by the Investigator.

Electronic patient files will be printed whenever the Monitor performs source data verification. Printouts must be signed and dated by the Investigator, countersigned by the Monitor and kept in a safe place at the site.

9.9 INVESTIGATOR SITE FILE AND ARCHIVING

The Investigator will be provided with an Investigator Site File upon initiation of the trial. This file will contain all documents necessary for the conduct of the trial and will be updated and completed throughout the trial. It must be available for review by the Monitor, and must be ready for Sponsor audit as well as for inspection by Health Authorities during and after the trial, and must be safely archived for at least 15 years (or per local requirements or as otherwise notified by the Sponsor) after the end of the trial. The documents to be thus archived include the Patient Identification List and the signed patient ICFs. If archiving of the Investigator Site File is no longer possible at the site, the Investigator must notify the Sponsor.

All original patient files (medical records) must be stored at the site (hospital, research institute, or practice) for the longest possible time permitted by the applicable regulations, and/or as per ICH GCP guidelines, whichever is longer. In any case, the Investigator should ensure that no destruction of medical records is performed without the written approval of the Sponsor.

9.10 MONITORING, QUALITY ASSURANCE AND INSPECTION BY HEALTH AUTHORITIES

This trial should be monitored in accordance with the ICH Note for Guidance on Good Clinical Practice (ICH Topic E6, 1996). The site Monitor will perform visits to the trial site at regular intervals.

Representatives of the Sponsor’s Quality Assurance unit or a designated organization, as well as Health Authorities, must be permitted to inspect all trial-related documents and other materials at the site, including the Investigator Site File, the completed eCRFs, Rebif®, and the patients’ original medical records/files.

The clinical trial protocol, each step of the data capture procedure, and the handling of the data, including the final clinical trial report, will be patient to independent Quality Assurance activities. Audits may be conducted at any time during or after the trial to ensure the validity and integrity of the trial data.

9.11 CHANGES TO THE CLINICAL TRIAL PROTOCOL

Changes to the clinical trial protocol will be documented in written protocol amendments. Major (substantial, significant) amendments will usually require submission to the Health Authorities and to the relevant IEC/IRB for approval or favorable opinion. In such cases, the amendment will be implemented only after approval or favorable opinion has been obtained.

Minor (non substantial) protocol amendments, including administrative changes, will be filed by the Sponsor and at the site. They will be submitted to the relevant IEC/IRB or to Health Authorities only where requested by pertinent regulations.

Any amendment that could have an impact on the patient’s agreement to participate in the trial requires the patient’s informed consent prior to implementation.

CLINICAL TRIAL REPORT AND PUBLICATION POLICY

9.12 CLINICAL TRIAL REPORT

After completion of the trial, a clinical trial report according to ICH Topic E3 will be written by the Sponsor in consultation with the Coordinating Investigator/ other relevant committees or groups.

9.13 PUBLICATION

The first publication will be a publication of the results of the analysis of the primary endpoint(s) that will include data from all trial sites.

The Investigator will inform the Sponsor in advance about any plans to publish or present data from the trial. Any publications and presentations of the results (abstracts in journals or newspapers, oral presentations, etc.), either in whole or in part, by Investigators or their representatives will require pre-submission review by the Sponsor.

The Sponsor will not suppress or veto publications, but maintains the right to delay publication in order to protect intellectual property rights.

10 REFERENCES

1. Ebers GC, Sadovnick AD. The role of genetic factors in multiple sclerosis susceptibility. J Neuroimmunol 1994; 54:1-17.
2. Sadovnick AD, Ebers GC, Dyment DA, Risch NJ. Evidence for genetic basis of multiple sclerosis. Lancet 1996; 347:1728-1730.
3. Sadovnick AD, Ebers GC, Dyment DA, Risch NJ, Canadian CSG. Evidence for the genetic basis of multiple sclerosis. Lancet 1996; 347:1728-1730.
4. Sadovnick AD, Ebers GC. Epidemiology in multiple sclerosis: A critical overview. Can J Neurol Sci 1993; 20:17-29.
5. Ota K, Matsui M, Milford EL, Mackin GA, Weiner HL, Hafler DA. T-cell recognition of an immunodominant myelin basic protein epitope in multiple sclerosis. Nature 1990; 346:183-187.
6. Martin R, McFarland HF, McFarlin DE. Immunological aspects of demyelinating diseases. Annu Rev Immunol 1992; 10:153-187.
7. Beck J, Rondot P, Catinot L, Falcoff E, Kirchner H, Wietzerbin J. Increased production of interferon-gamma and tumour necrosis factor precedes clinical manifestation in multiple sclerosis: Do cytokines trigger off exacerbations? Acta Neurol Scand 1988; 78:318-323.
8. Paty DW, Ebers GC. Clinical Features. In: Paty DW, Ebers GC, editors. Multiple Sclerosis. Philadelphia: I.A.Davis Company, 1998: 135-191.
9. Lublin FD, Reingold SC. Defining the clinical course of multiple sclerosis: Results of an international survey. Neurology 1996; 46:907-911
10. Runmarker B, Andersen O. Prognostic factors in a multiple sclerosis incidence cohort with twenty-five years of follow-up. Brain 1993; 116:117-134.
11. Gullevi Flensner. Fatique in relation to perceived health: people with multiple sclerosis compared with people in the general population, Journal compilation, 2008.
12. Paul Grossman. Mindfullness Training as an Intervention for Fibromyalgia: Evidence of Postintervention and 3-Year Follow-Up Benefits in Well-Being, Psychother Psychosom 2007; 76:226-233
13. Adherence to Long-term Therapies: Evidence for Action, WHO 2003, ISBN 92 4 154599 2. www.emro.who.int/ncd/Publications/adherence_report.pdf
14. AL-Sabbagh A, Bennet R, Kozma C, Dickson M, Meletiche DM. Medication gaps in disease-modifying drug therapy for multiple sclerosis are associated with an increased risk of relapse: findings from a national managed care database. Presented at the 18th meeting of the European Neurological Society, June 7-11, Nice, France. Poster P321.

15. Rio J, Porcel J, Tellez N, Sanchez-Betancourt A, Tintore M, Arevalo MJ, Nos C, Montalban X. Factors related with treatment adherence to interferon beta and glatiramer acetate therapy in multiple sclerosis. Mult Scler. 2005;11:306–309
16. Tremlett HL, Oger J, Interupted therapy; stopping and switching of the beta-interferons prescribed for MS. Neurology, 2003; 61: 551-554.
17. Cox D, Stone J, Managing self-injection difficulties in patients with relapsing remitting multiple sclerosis. J Neurosci Nurs 2006;38:167–171
18. Mohr DC, Boudewyn AC, Likosky W, Levine E, Goodkin D. Injectable medication for the treatment of multiple sclerosis: the influence of self-efficacy expectations and injection anxiety on adherence and ability to self-inject. Ann Behav Med. 2001;23:125–132.
19. Devonshire V, Lapierre Y, MacDonell R, Tello CR, Patti F, Fontoura P, Suchet L, Hyde R, Balla I, Kieseier BC, Frohman EM on behalf of the GAP Study Group. The Global Adherence Project (GAP)–A multicenter observational study on adherence to disease-modifying therapies in patients suffering from relapsing-remitting multiple sclerosis. Presented at the 22nd Congress of the European Committee for Treatment and Research in Multiple Sclerosis; September 27–30, 2006; Madrid, Spain
20. Treadaway K, Cutter G, Salter A, Lynch S, Simsarian J, Corboy J, Jeffery D, Cohen B, Mankowski K, Guarnaccia J, Schaeffer L, Kanter R, Brandes D, Kaufman C, Duncan D, Marder E, Allen A, Harney J, Cooper J, Woo D, Stüve O, Racke M, Frohman EM. Factors that influence adherence to disease modifying-therapy in multiple sclerosis. J Neurol. 2009 Apr;256(4):568-76
21. Cohen BA. Adherence to disease-modifying therapy for multiple sclerosis. Int J MS Care. 2006;(February Supplement):32–37.
22. Devonshire V, Cassidy D, Popelar L. Adherence to Disease-Modifying Therapy: Recognizing the Barriers and Offering Solutions. Ridgewood, NJ: Delaware Media Group, LLC; 2007.
23. Mohr DC, Goodkin DE, Likosky W *et al.* Therapeutic expectations of patients with multiple sclerosis upon initiating interferon beta-1b: relationship to adherence to treatment. *Mult Scler* 1996; 2: 222-6.
24. Kappos L. Traboulsee A. Constantinescu C. Eralinna JP. Forrestal F. Jongen P. Pollard J. Sandberg-Wollheim M. Sindic C. Stubinski B. Uitdehaag B. Li D. Long-term subcutaneous interferon beta-1a therapy in patients with relapsing-remitting MS. Neurology. 67(6):944-53, 2006 Sep 26.
25. McGuigan C, Hutchinson M. The multiple sclerosis impact scale (MSIS-29) is a reliable and sensitive measure. *J Neurosurg Psychiatry* 2004;75:266-269
26. Hobart J, Lamping D, Fitzpatrick R. Riazi A, Thompson A. The Multiple Sclerosis Impact Scale (MSIS-29). A new patient-based outcome measure. Brain (2001), 124, 962-973

27. Janssen MF, Birnie E, Haagsma JA, Bondel GJ. Comparing the standard EQ5D three-level system, with a five-level version. Value Health, 2008 Mar-Apr;11(2):275-84

28. Herrmann C. International Experiences with the Hospital Anxiety and Depression Scale – a review of validation data and clinical results. Journal of Psychosomatic Research, Vol. 42 No. 1 pp.17-41, 1997

29. MFISk JD, Ritvo PG, Ross L et al. Measuring the functional impact of fatigue: initial validation of the fatigue impact scale. Clin Infect Dis 1994;18(Suppl.1):S79-S83

30. Kostenius. C & Lindqvist. A-K. (2006). Health counselling. Hälsovägledning. Studentlitteratur.

31. Miller. W & Rollnick. S, (2010). Motivating conversation; to help individuals to change. Stockholm: Natur & Kultur.

Appendices

APPENDIX I- SPC ………………………………….

APPENDIX II- MSIS-29……………………………..

APPENDIX III- EQ5D-5L……………………………..

APPENDIX IV- HADS……………………………..

APPENDIX V- FSS……………………………..

APPENDIX VI- MFIS……………………………..

APPENDIX VII- MUSIQoL……………………………..
