# Supplementary material for: RebiQoL: A randomized trial of telemedicine patient support program for health-related quality of life and adherence in people with MS treated with Rebif
Source: PLoS One. 2019 Jul 5;14(7):e0218453. doi: 10.1371/journal.pone.0218453 (PMC6611587; doi:10.1371/journal.pone.0218453)
Supplement: S3 File — (PDF) [file pone.0218453.s003.pdf]

U.S. National Library of Medicine
ClinicalTrials.gov

A Phase 4 Study to Assess the Impact of Patient Support Program on Health Related Quality of Life and Adherence in Subjects With Relapsing-Remitting Multiple Sclerosis Administered Rebif® With the RebiSmart™ Device (RebiQoL)

The safety and scientific validity of this study is the responsibility of the study sponsor and investigators.
Listing a study does not mean it has been evaluated by the U.S. Federal Government. Read our disclaimer for details.

ClinicalTrials.gov Identifier: NCT01791244

Recruitment Status : Completed
First Posted : February 13, 2013
Results First Posted : July 12, 2017
Last Update Posted : August 24, 2017

Sponsor:
Merck KGaA, Darmstadt, Germany

Information provided by (Responsible Party):
Merck KGaA, Darmstadt, Germany

Study Details | Tabular View | **Study Results** | Disclaimer
How to Read a Study Record

**Study Type**	Interventional
**Study Design**	Allocation: Randomized; Intervention Model: Parallel Assignment; Masking: Single (Investigator); Primary Purpose: Treatment
**Conditions**	Multiple Sclerosis<br>Relapsing-Remitting
**Intervention**	Drug: Rebif®
**Enrollment**	93

Participant Flow

Go to

Recruitment Details	
Pre-assignment Details	A total of 93 subjects were randomized in the study out of which 46 were randomized to MinSupport Plus (MSP) and 47 to Technical support.

Arm/Group Title	Subject Support Program (MinSupport Plus)	Technical Support for the RebiSmart™ Device
Arm/Group Description	Subjects were administered Rebif® by the RebiSmart™ device at a dose of either 22 or 44 microgram (mcg) subcutaneously (SC) 3 times a week in accordance to the summary of product characteristics (SPC) along with subject support program MinSupport Plus which includes technical support for RebiSmart™ device, personal coaching regarding treatment and understanding of the disease, lifestyle guide and web support.	Subjects were administered Rebif® by the RebiSmart™ device at a dose of either 22 or 44 mcg SC 3 times a week in accordance to the SPC along with technical support for RebiSmart.
Period Title: **Overall Study**		
Started	46	47
Intent to Treat (ITT)	38	39
Completed	31	29
Not Completed	15	18
Reason Not Completed		
Adverse Event	5	7
Insufficient clinical response	10	10
Laboratory Abnormality	0	1

Baseline Characteristics

Go to

Arm/Group Title	Subject Support Program (MinSupport Plus)	Technical Support for the RebiSmart™ Device	Total
Arm/Group Description	Subjects were administered Rebif® by the RebiSmart™ device at a dose of	Subjects were administered Rebif® by the RebiSmart™ device at a dose of	Total of all reporting groups

		either 22 or 44 mcg SC 3 times a week in accordance to the SPC along with subject support program MinSupport Plus which includes technical support for RebiSmart™ device, personal coaching regarding treatment and understanding of the disease, lifestyle guide and web support.	either 22 or 44 mcg SC 3 times a week in accordance to the SPC along with technical support for RebiSmart.	
Overall Number of Baseline Participants		38	39	77
Baseline Analysis Population Description		The Intent-to-Treat (ITT) population included all subjects randomized into the trial and have completed at least 1 post-baseline assessment of the questionnaires.		
Age, Customized<br><br>Measure Type: Number<br>Unit of measure: Participants	Number Analyzed	38 participants	39 participants	77 participants
Between 18 to 72 years		38	39	77
Greater than (>) 72 years		0	0	0
Sex: Female, Male<br>Measure Type: Count of Participants				

Unit of measure: Participants				
	Number Analyzed	38 participants	39 participants	77 participants
	Female	24 63.2%	24 61.5%	48 62.3%
	Male	14 36.8%	15 38.5%	29 37.7%

Outcome Measures

Go to

1. Primary Outcome

Title	Change From Baseline in Multiple Sclerosis Impact Scale-29 (MSIS-29) Psychological Score at Month 12
Description	Multiple Sclerosis Impact Scale-29 (MSIS-29) is a validated MS specific questionnaire consisting of 29 questions of which 20 addressed the physical impact component and 9 assessed the psychological impact. A combined score can be generated, or both components can be reported separately. The psychological wellbeing assessment portion of the MSIS-29 was comprised of 9 questions in which subjects rate the impact of MS on their day-to-day life from 1=no impact to 5=extreme impact. The total Psychological Score was calculated using following formula: sum of score for 9 questions - 9/0.36. The total score range ranges from 0-100 where, lower total score indicates less psychologically-related impact while a higher total score indicates greater psychologically-related impact on a subject's functioning.
Time Frame	Baseline and Month 12

Outcome Measure Data

Analysis Population Description
The ITT population included all subjects randomized into the trial and have completed at least 1 post-baseline assessment of the questionnaires.

Arm/Group Title	Subject Support Program (MinSupport Plus)	Technical Support for the RebiSmart™ Device

Arm/Group Description:	Subjects were administered Rebif® by the RebiSmart™ device at a dose of either 22 or 44 mcg SC 3 times a week in accordance to the SPC along with subject support program MinSupport Plus which includes technical support for RebiSmart™ device, personal coaching regarding treatment and understanding of the disease, lifestyle guide and web support.	Subjects were administered Rebif® by the RebiSmart™ device at a dose of either 22 or 44 mcg SC 3 times a week in accordance to the SPC along with technical support for RebiSmart.
Overall Number of Participants Analyzed	38	39
Mean (Standard Deviation) Unit of Measure: units on a scale		
Baseline	35.19 (24.38)	30.48 (20.94)
Change at Month 12	-2.44 (19.38)	-3.04 (19.76)

Statistical Analysis 1

Statistical Analysis Overview	Comparison Group Selection	Subject Support Program (MinSupport Plus), Technical Support for the RebiSmart™ Device
	Comments	Linear mixed model, with baseline value, time, Expanded Disability Status Score (EDSS) at baseline and sex as fixed factors was used for the analysis.
	Type of Statistical Test	Superiority or Other
	Comments	[Not Specified]
Statistical Test of Hypothesis	P-Value	0.9148
	Comments	[Not Specified]
	Method	linear mixed model
	Comments	[Not Specified]
Method of	Estimation Parameter	Adjusted Mean Difference

Estimation	Estimated Value	0.48
	Confidence Interval	(2-Sided) 95%<br>-8.30 to 9.25
	Estimation Comments	[Not Specified]

2. Secondary Outcome

Title	Change From Baseline in Multiple Sclerosis Impact Scale-29 (MSIS-29) Psychological Score at Month 6
Description	Multiple Sclerosis Impact Scale-29 (MSIS-29) is a validated MS specific questionnaire consisting of 29 questions of which 20 addressed the physical impact component and 9 assessed the psychological impact. A combined score can be generated, or both components can be reported separately. The psychological wellbeing assessment portion of the MSIS-29 was comprised of 9 questions in which subjects rate the impact of MS on their day-to-day life from 1=no impact to 5=extreme impact. The total Psychological Score was calculated using following formula: sum of score for 9 questions - 9/0.36. The total score range ranges from 0-100 where, lower total score indicates less psychologically-related impact while a higher total score indicates greater psychologically-related impact on a subject's functioning.
Time Frame	Baseline and Month 6

Outcome Measure Data

Analysis Population Description
ITT population included all subjects randomized into the trial and have completed at least 1 post-baseline assessment of the questionnaires.

Arm/Group Title	Subject Support Program (MinSupport Plus)	Technical Support for the RebiSmart™ Device
Arm/Group Description:	Subjects were administered Rebif® by the RebiSmart™ device at a dose of either 22 or 44 mcg SC 3 times a week in accordance to the SPC along with subject support program MinSupport Plus which includes technical support for RebiSmart™ device, personal coaching regarding treatment and understanding of the disease, lifestyle guide and web support.	Subjects were administered Rebif® by the RebiSmart™ device at a dose of either 22 or 44 mcg SC 3 times a week in accordance to the SPC along with technical support for RebiSmart.

Overall Number of Participants Analyzed	38	39
Mean (Standard Deviation) Unit of Measure: units on a scale		
Baseline	35.19 (24.38)	30.48 (20.94)
Change at Month 6	-1.36 (16.82)	-1.59 (13.26)

3. Secondary Outcome

Title	Change From Baseline in Multiple Sclerosis Impact Scale-29 (MSIS-29) Total Score at Month 6 and 12
Description	Multiple Sclerosis Impact Scale-29 (MSIS-29) is a validated MS specific questionnaire consisting of 29 questions of which 20 addressed the physical impact component and 9 assessed the psychological impact. A combined score can be generated, or both components can be reported separately. The total score of the MSIS-29 was comprised of all the 29 questions in which subjects rate the impact of MS on their day-to-day life from 1=no impact to 5=extreme impact. The total Score was calculated using following formula: sum of score for 29 questions - 29/1.45. The total score range ranges from 0-100 where, lower total score indicates less psychologically-related impact while a higher total score indicates greater psychologically-related impact on a subject’s functioning.
Time Frame	Baseline, Month 6 and 12

Outcome Measure Data

Analysis Population Description
ITT population included all subjects randomized into the trial and have completed at least 1 post-baseline assessment of the questionnaires.

Arm/Group Title	Subject Support Program (MinSupport Plus)	Technical Support for the RebiSmart™ Device

Arm/Group Description:	Subjects were administered Rebif® by the RebiSmart™ device at a dose of either 22 or 44 mcg SC 3 times a week in accordance to the SPC along with subject support program MinSupport Plus which includes technical support for RebiSmart™ device, personal coaching regarding treatment and understanding of the disease, lifestyle guide and web support.	Subjects were administered Rebif® by the RebiSmart™ device at a dose of either 22 or 44 mcg SC 3 times a week in accordance to the SPC along with technical support for RebiSmart.
Overall Number of Participants Analyzed	38	39
Mean (Standard Deviation) Unit of Measure: units on a scale		
Baseline	21.81 (21.55)	18.01 (16.61)
Change at Month 6	0.56 (9.35)	0.14 (11.19)
Change at Month 12	1.00 (11.34)	0.00 (10.45)

4. Secondary Outcome

Title	Change From Baseline in Euro Quality of Life Questionnaire With 5 Questions Alternatives (EQ5D-5L) Summary Score at Month 6 and 12
Description	Quality of life was assessed using the EQ5D-5L score, which is one of the most widely used generic index measures of health-related quality of life. It consists of a 5-item descriptive system that measures 5 dimensions of health, including mobility, self-care, usual activities, pain/discomfort, and anxiety/depression. Each dimension comprises 5 levels with corresponding numeric scores ranging from 1 (no problems) through 5 (extreme problems) in which 1= no problems, 2= slight problems, 3= moderate problems, 4= severe problems, and 5= extreme problems. A unique EQ5D-5L health state was defined by combining the numeric level scores for each of the 5 dimensions and the total score ranges from 5 to 25. An increase in the EQ5D-5L total score indicates worsening.

Time Frame	Baseline, Month 6 and 12

Outcome Measure Data

Analysis Population Description

ITT population included all subjects randomized into the trial and have completed at least 1 post-baseline assessment of the questionnaires.

Arm/Group Title	Subject Support Program (MinSupport Plus)	Technical Support for the RebiSmart™ Device
Arm/Group Description:	Subjects were administered Rebif® by the RebiSmart™ device at a dose of either 22 or 44 mcg SC 3 times a week in accordance to the SPC along with subject support program MinSupport Plus which includes technical support for RebiSmart™ device, personal coaching regarding treatment and understanding of the disease, lifestyle guide and web support.	Subjects were administered Rebif® by the RebiSmart™ device at a dose of either 22 or 44 mcg SC 3 times a week in accordance to the SPC along with technical support for RebiSmart.
Overall Number of Participants Analyzed	38	39
Mean (Standard Deviation) Unit of Measure: units on a scale		
Baseline	8.84 (3.37)	8.03 (2.30)
Change at Month 6	0.18 (2.17)	0.10 (1.76)
Change at Month 12	0.05 (2.51)	-0.08 (1.86)

5. Secondary Outcome

Title	Change From Baseline in Euro Quality of Life Questionnaire With 5 Questions Alternatives (EQ5D-5L) Visual Analogue Scale (VAS) Scale at Month 6 and 12
Description	EQ-5D-5L VAS was used to record a subject’s rating for his/her current health-

	related quality of life state and captured on a vertical VAS (0-100), where 0 = worst imaginable health state and 100 = best imaginable health state.
Time Frame	Baseline, Month 6 and 12

Outcome Measure Data

Analysis Population Description

ITT population included all subjects randomized into the trial and have completed at least 1 post-baseline assessment of the questionnaires.

Arm/Group Title	Patient Support Program (MinSupport Plus)	Technical Support for the RebiSmart™ Device
Arm/Group Description:	Subjects were administered Rebif® by the RebiSmart™ device at a dose of either 22 or 44 mcg SC 3 times a week in accordance to the SPC along with subject support program MinSupport Plus which includes technical support for RebiSmart™ device, personal coaching regarding treatment and understanding of the disease, lifestyle guide and web support.	Subjects were administered Rebif® by the RebiSmart™ device at a dose of either 22 or 44 mcg SC 3 times a week in accordance to the SPC along with technical support for RebiSmart.
Overall Number of Participants Analyzed	38	39
Mean (Standard Deviation) Unit of Measure: units on a scale		
Baseline	71.18 (20.70)	71.49 (16.61)
Change at Month 6	-0.71 (11.36)	-3.38 (19.35)
Change at Month 12	-0.84 (16.07)	-2.31 (20.60)

6. Secondary Outcome

Title	Percentage of Subjects With Treatment Adherence at Month 6 and 12

Description	According to the World Health Organisation (WHO), treatment adherence is defined as both compliance (taking the medication in the correct dose and according to the schedule prescribed) and persistency (maintenance of the drug regimen over the long-term). Percentage of subjects with <10% missed injections (measured with the software RDS 2.0) during 6 and 12 months were reported.
Time Frame	Month 6 and 12

Outcome Measure Data

Analysis Population Description

ITT population included all subjects randomized into the trial and have completed at least 1 post-baseline assessment of the questionnaires.

Arm/Group Title	Subject Support Program (MinSupport Plus)	Technical Support for the RebiSmart™ Device
Arm/Group Description:	Subjects were administered Rebif® by the RebiSmart™ device at a dose of either 22 or 44 mcg SC 3 times a week in accordance to the SPC along with subject support program MinSupport Plus which includes technical support for RebiSmart™ device, personal coaching regarding treatment and understanding of the disease, lifestyle guide and web support.	Subjects were administered Rebif® by the RebiSmart™ device at a dose of either 22 or 44 mcg SC 3 times a week in accordance to the SPC along with technical support for RebiSmart.
Overall Number of Participants Analyzed	38	39
Measure Type: Number Unit of Measure: Percentage of Subjects		
At Month 6	71.05	64.10
At Month 12	65.79	53.85

7. Secondary Outcome

Title	Change From Baseline in Fatigue Severity Scale (FSS) Score at Month 6 and

	12
Description	Fatigue Severity Scale (FSS) is a method of evaluating fatigue in multiple sclerosis and is designed to differentiate fatigue from clinical depression, since both share some of the same symptoms. The Fatigue Severity Scale is a 9-item questionnaire developed to assess the level of fatigue due to neurological disease, were each assessed on a 1-7 scale (1= no fatigue and 7= severe fatigue). The total score was calculated as the average of individual 9-items and ranged from 1 to 7 with a higher value indicating greater impairment due to fatigue.
Time Frame	Baseline, Month 6 and 12

Outcome Measure Data

Analysis Population Description
ITT population included all subjects randomized into the trial and have completed at least 1 post-baseline assessment of the questionnaires.

Arm/Group Title	Subject Support Program (MinSupport Plus)	Technical Support for the RebiSmart™ Device
Arm/Group Description:	Subjects were administered Rebif® by the RebiSmart™ device at a dose of either 22 or 44 mcg SC 3 times a week in accordance to the SPC along with subject support program MinSupport Plus which includes technical support for RebiSmart™ device, personal coaching regarding treatment and understanding of the disease, lifestyle guide and web support.	Subjects were administered Rebif® by the RebiSmart™ device at a dose of either 22 or 44 mcg SC 3 times a week in accordance to the SPC along with technical support for RebiSmart.
Overall Number of Participants Analyzed	38	39
Mean (Standard Deviation) Unit of Measure: units on a scale		
Baseline	3.93 (1.75)	3.98 (1.72)

Change at Month 6	0.15 (0.79)	0.05 (0.81)
Change at Month 12	0.13 (0.89)	0.08 (1.09)

8. Secondary Outcome

Title	Change From Baseline in Modified Fatigue Impact Scale Score at Month 6 and 12
Description	The Modified Fatigue Impact Scale is a list of 21 statements describing how fatigue may affect a person's functioning. Answers ranging from 0 (Never) to 4 (Almost always). A total score ranged from a possible 0 (no fatigue impact) to 84 (almost always impacted by fatigue). A lower total score indicates less fatigue-related impact while a higher total score indicates greater fatigue-related impact on a subject's functioning.
Time Frame	Baseline, Month 6 and 12

Outcome Measure Data

Analysis Population Description
ITT population included all subjects randomized into the trial and have completed at least 1 post-baseline assessment of the questionnaires.. Here, "Overall Number of subjects analyzed" signifies those subjects who were evaluable for this outcome Measure.

Arm/Group Title	Subject Support Program (MinSupport Plus)	Technical Support for the RebiSmart™ Device
Arm/Group Description:	Subjects were administered Rebif® by the RebiSmart™ device at a dose of either 22 or 44 microgram (mcg) subcutaneously (SC) 3 times a week in accordance to the summary of product characteristics (SPC) along with subject support program MinSupport Plus which includes technical support for RebiSmart™ device, personal coaching regarding treatment and understanding of the disease, lifestyle guide and web support.	Subjects were administered Rebif® by the RebiSmart™ device at a dose of either 22 or 44 mcg SC 3 times a week in accordance to the SPC along with technical support for RebiSmart.
Overall Number of Participants Analyzed	37	36

Mean (Standard Deviation) Unit of Measure: units on a scale		
Baseline	29.74 (20.50)	31.08 (19.08)
Change at Month 6	3.89 (10.81)	2.27 (9.78)
Change at Month 12	3.75 (10.43)	1.78 (10.65)

9. Secondary Outcome

Title	Change From Baseline in Modified Fatigue Impact Scale Index at Month 6 and 12
Description	The Modified Fatigue Impact Index assesses fatigue- severity, distress, or degree of interference. Modified Fatigue Impact Scale Index was expressed in terms of percentage and ranged from 0% (no fatigue) to 100% (almost always impacted by fatigue).
Time Frame	Baseline, Month 6 and 12

Outcome Measure Data

Analysis Population Description

ITT population included all subjects randomized into the trial and have completed at least 1 post-baseline assessment of the questionnaires. Here, “Number of subjects analyzed” signifies those subjects who were evaluable for this outcome Measure.

Arm/Group Title	Patient Support Program (MinSupport Plus)	Technical Support for the RebiSmart™ Device
Arm/Group Description:	Subjects were administered Rebif® by the RebiSmart™ device at a dose of either 22 or 44 mcg SC 3 times a week in accordance to the SPC along with subject support program MinSupport Plus which includes technical support for RebiSmart™ device, personal coaching regarding treatment and understanding of the disease, lifestyle guide and web support.	Subjects were administered Rebif® by the RebiSmart™ device at a dose of either 22 or 44 mcg SC 3 times a week in accordance to the SPC along with technical support for RebiSmart.

Overall Number of Participants Analyzed	37	36
Mean (Standard Deviation) Unit of Measure: Percentage of fatigue		
Baseline	35.40 (24.41)	37.00 (22.72)
Change at Month 6	4.63 (12.87)	2.71 (11.64)
Change at Month 12	4.47 (12.42)	2.12 (12.68)

10. Secondary Outcome

Title	Change From Baseline in Hospital Anxiety and Depression Scale (HADS) Score at Month 6 and 12
Description	Hospital Anxiety and Depression Scale (HADS) was used to measure depression and anxiety in patients. The scale was limited to 14 questions, a practical tool for identifying and quantifying the two most common forms psychological disturbances in medical subjects. 7 of the items relate to anxiety and 7 relate to depression. Each item on the questionnaire was scored from 0-3 giving a total score between 0 and 21 for either anxiety or depression where higher score indicates more anxiety/depression.
Time Frame	Baseline, Month 6 and 12

Outcome Measure Data

Analysis Population Description
ITT population included all subjects randomized into the trial and have completed at least 1 post-baseline assessment of the questionnaires.

Arm/Group Title	Subject Support Program (MinSupport Plus)	Technical Support for the RebiSmart™ Device

Arm/Group Description:	Subjects were administered Rebif® by the RebiSmart™ device at a dose of either 22 or 44 mcg SC 3 times a week in accordance to the SPC along with subject support program MinSupport Plus which includes technical support for RebiSmart™ device, personal coaching regarding treatment and understanding of the disease, lifestyle guide and web support.	Subjects were administered Rebif® by the RebiSmart™ device at a dose of either 22 or 44 mcg SC 3 times a week in accordance to the SPC along with technical support for RebiSmart.
Overall Number of Participants Analyzed	38	39
Mean (Standard Deviation) Unit of Measure: units on a scale		
Baseline (Anxiety Score)	6.13 (4.14)	5.62 (3.21)
Change at Month 6 (Anxiety Score)	-0.21 (2.92)	-0.56 (2.78)
Change at Month 12 (Anxiety Score)	-0.42 (3.48)	-0.18 (3.26)
Baseline (Depression Score)	4.26 (3.49)	3.72 (2.77)
Change at Month 6 (Depression Score)	0.61 (2.43)	0.46 (2.85)

Change at Month 12 (Depression Score)	0.42 (3.61)	0.74 (3.14)

11. Secondary Outcome

Title	Number of Subjects With Working Ability at Month 12
Description	Working ability was assessed by measuring the number of subjects for the following categories: 1) Subjects with full sickness/disability pension, 2) Subjects who were employed or had their own business, 3) Subjects who were retired, 4) Subjects who were studying, 5) None of the above.
Time Frame	Month 12

Outcome Measure Data

Analysis Population Description
ITT population included all subjects randomized into the trial and have completed at least 1 post-baseline assessment of the questionnaires. Here, “Overall Number of subjects analyzed” signifies those subjects who were evaluable for this outcome Measure.

Arm/Group Title	Subject Support Program (MinSupport Plus)	Technical Support for the RebiSmart™ Device
Arm/Group Description:	Subjects were administered Rebif® by the RebiSmart™ device at a dose of either 22 or 44 mcg SC 3 times a week in accordance to the SPC along with subject support program MinSupport Plus which includes technical support for RebiSmart™ device, personal coaching regarding treatment and understanding of the disease, lifestyle guide and web support.	Subjects were administered Rebif® by the RebiSmart™ device at a dose of either 22 or 44 mcg SC 3 times a week in accordance to the SPC along with technical support for RebiSmart.
Overall Number of Participants Analyzed	32	35
Measure Type: Number<br>Unit of Measure: Subjects		

Subjects with full sickness/disability pension	4	5
Subjects who were employed or had own business	22	25
Subject who were retired	2	2
Subject who were studying	3	2
None of the above	1	1

12. Secondary Outcome

Title	Percentage of Subjects With Adverse Events (AE) up to Month 12
Description	AE was defined as any untoward medical occurrence which does not necessarily have a causal relationship with this the study drug. An AE was defined as any unfavourable and unintended sign (including an abnormal laboratory finding), symptom, or disease temporally associated with the use of study drug, whether or not considered related to the study drug. A serious AE was an AE that resulted in any of the following outcomes: death; life threatening; persistent/significant disability/incapacity; initial or prolonged inpatient hospitalization; congenital anomaly/birth defect or was otherwise considered medically important. Treatment Emergent Adverse Events (TEAEs) include both Serious TEAEs and non-serious TEAEs.
Time Frame	Baseline up to Month 12

Outcome Measure Data

Analysis Population Description
The Safety Population included all subjects who were randomized and received at least 1 dose of trial treatment.

Arm/Group Title	Subject Support Program (MinSupport Plus)	Technical Support for the RebiSmart™ Device

Arm/Group Description:	Subjects were administered Rebif® by the RebiSmart™ device at a dose of either 22 or 44 mcg SC 3 times a week in accordance to the SPC along with subject support program MinSupport Plus which includes technical support for RebiSmart™ device, personal coaching regarding treatment and understanding of the disease, lifestyle guide and web support.	Subjects were administered Rebif® by the RebiSmart™ device at a dose of either 22 or 44 mcg SC 3 times a week in accordance to the SPC along with technical support for RebiSmart.
Overall Number of Participants Analyzed	46	47
Measure Type: Number Unit of Measure: Percentage of Subjects		
	45.7	61.7

13. Secondary Outcome

Title	Number of Subjects With Response Based on Lifestyle Questionnaire for (MinSupport Plus) at Month 6 and 12
Description	Lifestyle Questionnaire was used to assess the quality of life for subjects based on following parameters: Stress, Alcohol, Cost, Physical Aspect, Sleep, Activity and Smoking. Subjects provided their responses on the basis of three color codes: Green, Orange and Red, where Green refers to – no problem; Orange refers to – some problem and red refers to – definite/debilitating problem.
Time Frame	Month 6 and 12

Outcome Measure Data

Analysis Population Description
ITT population was used. Here, “Overall Number of Participants Analyzed” signifies those subjects who were evaluable for this outcome Measure and “n” signifies those subjects who were evaluable for specified time points, respectively.

Arm/Group Title	Subject Support Program (MinSupport Plus)
Arm/Group Description:	Subjects were administered Rebif® by the RebiSmart™ device at a dose of either 22 or 44 mcg SC 3 times a week in accordance to the SPC along with subject support program MinSupport Plus which includes technical support for RebiSmart™ device, personal coaching regarding treatment and understanding of the disease, lifestyle guide and web support.
Overall Number of Participants Analyzed	32
Measure Type: Number Unit of Measure: Subjects	
Month 6: Stress: Green; n= 30	2
Month 6: Stress: Orange; n= 30	3
Month 6: Stress: Red; n= 30	3
Month 12: Stress: Green; n= 31	3
Month 12: Stress: Orange; n= 31	2
Month 12: Stress: Red; n= 31	2
Month 6: Alcohol: Green; n= 30	7

Month 6: Alcohol: Orange; n= 30	1
Month 6: Alcohol: Red; n= 30	1
Month 12: Alcohol: Green; n= 32	6
Month 12: Alcohol: Orange; n= 32	6
Month 12: Alcohol: Red; n= 32	6
Month 6: Cost: Green; n= 30	4
Month 6: Cost: Orange; n= 30	4
Month 6: Cost: Red; n= 30	4
Month 12: Cost: Green; n= 32	3
Month 12: Cost: Orange; n= 32	3
Month 12: Cost: Red; n= 32	3
Month 6: Physical: Green; n= 30	5

Month 6: Physical: Orange; n= 30	2
Month 6: Physical: Red; n= 30	1
Month 12: Physical: Green; n= 32	5
Month 12: Physical: Orange; n= 32	1
Month 12: Physical: Red; n= 32	1
Month 6: Activity: Green; n= 30	5
Month 6: Activity: Orange; n= 30	2
Month 6: Activity: Red; n= 30	1
Month 12: Activity: Green; n= 32	5
Month 12: Activity: Orange; n= 32	1
Month 12: Activity: Red; n= 32	1
Month 6: Sleep: Green; n= 30	3

Month 6: Sleep: Orange; n= 30	4
Month 6: Sleep: Red; n= 30	1
Month 12: Sleep: Green; n= 32	3
Month 12: Sleep: Orange; n= 32	2
Month 12: Sleep: Red; n= 32	1
Month 6: Smoking: Green; n= 31	5
Month 6: Smoking: Orange; n= 31	1
Month 6: Smoking: Red; n= 31	1
Month 12: Smoking: Green; n= 32	5
Month 12: Smoking: Orange; n= 32	1
Month 12: Smoking: Red; n= 32	1

14. Secondary Outcome

Title	Number of Subjects With Response Based on Subject Satisfaction Questionnaire at Month 12

Description	The subject satisfaction questionnaire was defined as satisfaction with overall treatment and support from health care providers during the last 12 months. Subjects were asked to rate their satisfaction by choosing either "Very discontented, discontented, contented or Very contented".
Time Frame	Month 12

Outcome Measure Data

Analysis Population Description

ITT population included all subjects randomized into the trial and have completed at least 1 post-baseline assessment of the questionnaires. Here, "Number of Subjects Analyzed" signifies those subjects who were evaluable for this outcome measure.

Arm/Group Title	Subject Support Program (MinSupport Plus)	Technical Support for the RebiSmart™ Device
Arm/Group Description:	Subjects were administered Rebif® by the RebiSmart™ device at a dose of either 22 or 44 mcg SC 3 times a week in accordance to the SPC along with subject support program MinSupport Plus which includes technical support for RebiSmart™ device, personal coaching regarding treatment and understanding of the disease, lifestyle guide and web support.	Subjects were administered Rebif® by the RebiSmart™ device at a dose of either 22 or 44 mcg SC 3 times a week in accordance to the SPC along with technical support for RebiSmart.
Overall Number of Participants Analyzed	30	33
Measure Type: Number Unit of Measure: Subjects		
Very discontented	1	2
Discontented	6	5
Contented	13	11
Very contented	10	15

15. Secondary Outcome

Title	Number of Subjects With Response Based on Health Care Personnel Satisfaction Questionnaire at Month 12
Description	The subject satisfaction questionnaire was defined as satisfaction with overall treatment and support from health care providers during the last 12 months. Subjects were asked to rate their satisfaction by choosing either “Very unsatisfied, unsatisfied, satisfied or very satisfied”.
Time Frame	Month 12

Outcome Measure Data

Analysis Population Description

ITT population included all subjects randomized into the trial and have completed at least 1 post-baseline assessment of the questionnaires. Here, "Overall Number of Participants Analyzed" signifies those subjects who were evaluable for this outcome measure.

Arm/Group Title	Subject Support Program (MinSupport Plus)	Technical Support for the RebiSmart™ Device
Arm/Group Description:	Subjects were administered Rebif® by the RebiSmart™ device at a dose of either 22 or 44 mcg SC 3 times a week in accordance to the SPC along with subject support program MinSupport Plus which includes technical support for RebiSmart™ device, personal coaching regarding treatment and understanding of the disease, lifestyle guide and web support.	Subjects were administered Rebif® by the RebiSmart™ device at a dose of either 22 or 44 mcg SC 3 times a week in accordance to the SPC along with technical support for RebiSmart.
Overall Number of Participants Analyzed	18	13
Measure Type: Number Unit of Measure: Subjects		
Very unsatisfied	0	1
Unsatisfied	2	0
Satisfied	10	9

Very satisfied	6	3

16. Secondary Outcome

Title	Number of Subjects With Lifestyle Goals for MinSupport Plus at Month 12
Description	Subjects defined up to 4 personal lifestyle goals during the first study week. Subjects completed the following questions related to lifestyle goals achieved during this study: 1. Was the goal achieved? (Yes/No) 2. If yes, better than expected or achieved as expected? 3. If better than expected, a lot or a little better than expected? 4. If no, a little or a lot less than expected?
Time Frame	Month 12

Outcome Measure Data

Analysis Population Description

ITT population included all subjects randomized into the trial and have completed at least 1 post-baseline assessment of the questionnaires. Here, "n" signifies subjects who were evaluable for the specific goal in this outcome measure.

Arm/Group Title	Subject Support Program (MinSupport Plus)
Arm/Group Description:	Subjects were administered Rebif® by the RebiSmart™ device at a dose of either 22 or 44 mcg SC 3 times a week in accordance to the SPC along with subject support program MinSupport Plus which includes technical support for RebiSmart™ device, personal coaching regarding treatment and understanding of the disease, lifestyle guide and web support.
Overall Number of Participants Analyzed	38
Measure Type: Number<br>Unit of Measure: Subjects	
Goal 1(G1) : Goal Achieved; n= 38	18
G1: Goal not Achieved; n= 38	4
G1: Missing; n= 38	16
G1 Achieved: Better than Expected; n= 18	5

G1 Achieved: As Expected; n= 18	10
G1 Achieved: Missing; n= 18	3
G1Acheived:Better than Expected:Little Better;n= 5	4
G1Achieved:Better than expected:Lot better; n= 5	0
G1Achieved:Better than Expected: Missing; n= 5	1
G1 not Achieved: Little Less; n= 4	1
G1 not Achieved: Lot Less; n= 4	0
G1 not Achieved: Missing; n= 4	3
Goal 2 (G2): Goal Achieved; n= 38	3
G2: Goal not Achieved; n= 38	1
G2: Missing; n= 38	34
G2 Achieved: Better than Expected; n= 3	2
G2 Achieved: As Expected; n= 3	0
G2 Achieved: Missing; n= 3	1
G2Achieved:Better than Expected:Little Better;n= 2	1
G2 Achieved: Better than Expected:Lot Better; n= 2	1

G2 Achieved: Better than Expected: Missing; n= 2	0
G2 not Achieved: Little Less; n= 1	0
G2 not Achieved: Lot Less; n= 1	1
G2 not Achieved: Missing; n= 1	0
Goal 3 (G3): Goal Achieved; n= 38	0
G3: Goal not Achieved; n= 38	1
G3: Missing; n= 38	37
G3 Achieved: As Expected; n= 0	0
G3 Achieved: Better than Expected; n= 0	0
G3 Achieved: Missing; n= 0	0
G3 Achieved:Better than Expected:Little Better;n=0	0
G3 Achieved:Better than Expected: Lot Better;n=0	0
G3 Achieved: Better than Expected: Missing; n= 0	0
G3 not Achieved: Little Less; n= 1	1
G3 not Achieved: Lot Less; n= 1	0
G3 not Achieved: Missing; n= 1	0

Goal 4 (G4): Goal Achieved; n= 38	0
G4: Goal not Achieved; n= 38	1
G4: Missing; n= 38	37
G4 Achieved: Better than Expected; n= 0	0
G4 Achieved: As Expected; n= 0	0
G4 Achieved: Missing; n= 0	0
G4 Expected:Little Better; n= 0	0
G4Achieved:Better than expected:Lot Better; n= 0	0
G4 Achieved: Better than Expected: Missing; n= 0	0
G4 not Achieved: Little Less; n= 1	0
G4 not Achieved: Lot Less; n= 1	0
G4 not Achieved: Missing; n= 1	1

Adverse Events

Go to

Time Frame	Baseline up to Month 12
Adverse Event Reporting Description	[Not Specified]

Arm/Group Title	Patient Support Program (MinSupport Plus)	Technical Support for the RebiSmart™ Device
Arm/Group Description	Subjects were administered Rebif® by the RebiSmart™ device at a dose of either 22 or 44 mcg SC 3 times a week	Subjects were administered Rebif® by the RebiSmart™ device at a dose of either 22 or 44 mcg SC 3 times a week

	in accordance to the SPC along with subject support program MinSupport Plus which includes technical support for RebiSmart™ device, personal coaching regarding treatment and understanding of the disease, lifestyle guide and web support.	in accordance to the SPC along with technical support for RebiSmart.

All-Cause Mortality

	Patient Support Program (MinSupport Plus)	Technical Support for the RebiSmart™ Device
	Affected / at Risk (%)	Affected / at Risk (%)
Total	--/--	--/--

Serious Adverse Events

	Patient Support Program (MinSupport Plus)	Technical Support for the RebiSmart™ Device
	Affected / at Risk (%)	Affected / at Risk (%)
Total	2/46 (4.35%)	0/47 (0.00%)
Infections and infestations		
Pneumonia * 1	1/46 (2.17%)	0/47 (0.00%)
Urinary tract infection * 1	2/46 (4.35%)	0/47 (0.00%)

* Indicates events were collected by non-systematic assessment

1 Term from vocabulary, MedDRA 16.0

Other (Not Including Serious) Adverse Events

Frequency Threshold for Reporting Other Adverse Events	0%

	Patient Support Program (MinSupport Plus)	Technical Support for the RebiSmart™ Device
	Affected / at Risk (%)	Affected / at Risk (%)
Total	19/46 (41.30%)	29/47 (61.70%)
Blood and lymphatic system disorders		
Leukopenia * 1	0/46 (0.00%)	1/47 (2.13%)
Cardiac disorders		

Palpitations * 1	0/46 (0.00%)	1/47 (2.13%)
Ear and labyrinth disorders		
Vertigo * 1	0/46 (0.00%)	1/47 (2.13%)
Eye disorders		
Retinal disorder * 1	1/46 (2.17%)	0/47 (0.00%)
Gastrointestinal disorders		
Aphthous stomatitis * 1	0/46 (0.00%)	1/47 (2.13%)
Constipation * 1	0/46 (0.00%)	1/47 (2.13%)
Diarrhoea * 1	1/46 (2.17%)	0/47 (0.00%)
Gastritis * 1	1/46 (2.17%)	0/47 (0.00%)
Vomiting * 1	1/46 (2.17%)	0/47 (0.00%)
General disorders		
Administration site pain * 1	2/46 (4.35%)	0/47 (0.00%)
Chills * 1	1/46 (2.17%)	1/47 (2.13%)
Fatigue * 1	2/46 (4.35%)	1/47 (2.13%)
Influenza like illness * 1	2/46 (4.35%)	2/47 (4.26%)
Injection site pain * 1	1/46 (2.17%)	0/47 (0.00%)
Injection site reaction * 1	0/46 (0.00%)	1/47 (2.13%)
Pain * 1	1/46 (2.17%)	1/47 (2.13%)
Pyrexia * 1	1/46 (2.17%)	1/47 (2.13%)
Infections and infestations		
Nasopharyngitis * 1	2/46 (4.35%)	1/47 (2.13%)
Pneumonia * 1	1/46 (2.17%)	0/47 (0.00%)
Upper respiratory tract infection * 1	0/46 (0.00%)	1/47 (2.13%)
Urinary tract infection * 1	2/46 (4.35%)	0/47 (0.00%)
Injury, poisoning and procedural complications		
Injection related reaction * 1	1/46 (2.17%)	0/47 (0.00%)
Investigations		
Hepatic enzyme increased * 1	1/46 (2.17%)	5/47 (10.64%)
Neutralising antibodies * 1	1/46 (2.17%)	1/47 (2.13%)
Musculoskeletal and connective tissue disorders		
Muscular weakness * 1	0/46 (0.00%)	1/47 (2.13%)
Musculoskeletal stiffness * 1	1/46 (2.17%)	0/47 (0.00%)
Neoplasms benign, malignant and unspecified (incl cysts and polyps)		

Lymphoma *1	0/46 (0.00%)	1/47 (2.13%)
Nervous system disorders		
Dizziness *1	1/46 (2.17%)	0/47 (0.00%)
Headache *1	2/46 (4.35%)	3/47 (6.38%)
Hemiparesis *1	0/46 (0.00%)	1/47 (2.13%)
Hypertonia *1	0/46 (0.00%)	1/47 (2.13%)
Hypoaesthesia *1	0/46 (0.00%)	1/47 (2.13%)
Migraine *1	0/46 (0.00%)	1/47 (2.13%)
Multiple sclerosis *1	1/46 (2.17%)	1/47 (2.13%)
Multiple sclerosis relapse *1	2/46 (4.35%)	2/47 (4.26%)
Neuralgia *1	0/46 (0.00%)	1/47 (2.13%)
Paraesthesia *1	1/46 (2.17%)	0/47 (0.00%)
Visual field defect *1	0/46 (0.00%)	1/47 (2.13%)
Psychiatric disorders		
Depression *1	1/46 (2.17%)	2/47 (4.26%)
Fear of needles *1	1/46 (2.17%)	0/47 (0.00%)
Insomnia *1	2/46 (4.35%)	0/47 (0.00%)
Mood swings *1	1/46 (2.17%)	2/47 (4.26%)
Skin and subcutaneous tissue disorders		
Eczema *1	1/46 (2.17%)	0/47 (0.00%)
Psoriasis *1	0/46 (0.00%)	1/47 (2.13%)
Rash *1	0/46 (0.00%)	2/47 (4.26%)
Vascular disorders		
Hypertension *1	0/46 (0.00%)	1/47 (2.13%)

* Indicates events were collected by non-systematic assessment
1 Term from vocabulary, MedDRA 16.0

Limitations and Caveats

Go to

[Not Specified]

More Information

Go to

Certain Agreements

Principal Investigators are NOT employed by the organization sponsoring the study.

There is NOT an agreement between Principal Investigators and the Sponsor (or its agents) that restricts the PI's rights to discuss or publish trial results after the trial is completed.

Results Point of Contact

Name/Title: Merck KGaA Communication Center
Organization: Merck Healthcare, a business of Merck KGaA, Darmstadt, Germany
Phone: +49-6151-72-5200
EMail: service@merckgroup.com

Responsible Party:	Merck KGaA, Darmstadt, Germany
ClinicalTrials.gov Identifier:	NCT01791244 History of Changes
Other Study ID Numbers:	EMR 200136-560<br>2012-004887-22 (EudraCT Number)
First Submitted:	February 12, 2013
First Posted:	February 13, 2013
Results First Submitted:	April 19, 2017
Results First Posted:	July 12, 2017
Last Update Posted:	August 24, 2017
